# Initial eye gaze to faces and its functional consequence on face identification abilities in autism spectrum disorder

**DOI:** 10.1186/s11689-019-9303-z

**Published:** 2019-12-28

**Authors:** Kimberly B. Schauder, Woon Ju Park, Yuliy Tsank, Miguel P. Eckstein, Duje Tadin, Loisa Bennetto

**Affiliations:** 1 Department of Psychology, University of Rochester, Meliora Hall, P.O Box 270266, Rochester, NY 14627 USA; 2 Center for Visual Science, University of Rochester, Rochester, USA; 3 Children’s National Medical Center, Center for Autism Spectrum Disorders, 15245 Shady Grove Road Suite 350, Rockville, MD 20850 USA; 4 Department of Psychology, University of Washington, Guthrie Hall Box 351525, Seattle, WA 98195 USA; 5 Department of Psychological and Brain Sciences, University of California Santa Barbara, Santa Barbara, CA 93106-9660 USA; 6 Department of Brain and Cognitive Sciences, University of Rochester, 310 Meliora Hall, P.O Box 270268, Rochester, NY 14627-0268 USA; 7 Departments of Ophthalmology and Neuroscience, University of Rochester School of Medicine, Rochester, USA

**Keywords:** Autism spectrum disorder, Face identification, Eye gaze

## Abstract

**Background:**

Autism spectrum disorder (ASD) is a neurodevelopmental disorder defined and diagnosed by core deficits in social communication and the presence of restricted and repetitive behaviors. Research on face processing suggests deficits in this domain in ASD but includes many mixed findings regarding the nature and extent of these differences. The first eye movement to a face has been shown to be highly informative and sufficient to achieve high performance in face identification in neurotypical adults. The current study focused on this critical moment shown to be essential in the process of face identification.

**Methods:**

We applied an established eye-tracking and face identification paradigm to comprehensively characterize the initial eye movement to a face and test its functional consequence on face identification performance in adolescents with and without ASD (*n* = 21 per group), and in neurotypical adults. Specifically, we presented a series of faces and measured the landing location of the first saccade to each face, while simultaneously measuring their face identification abilities. Then, individuals were guided to look at specific locations on the face, and we measured how face identification performance varied as a function of that location. Adolescent participants also completed a more traditional measure of face identification which allowed us to more fully characterize face identification abilities in ASD.

**Results:**

Our results indicate that the location of the initial look to faces and face identification performance for briefly presented faces are intact in ASD, ruling out the possibility that deficits in face perception, at least in adolescents with ASD, begin with the initial eye movement to the face. However, individuals with ASD showed impairments on the more traditional measure of face identification.

**Conclusion:**

Together, the observed dissociation between initial, rapid face perception processes, and other measures of face perception offers new insights and hypotheses related to the timing and perceptual complexity of face processing and how these specific aspects of face identification may be disrupted in ASD.

## Background

Autism spectrum disorder (ASD) is a neurodevelopmental disorder defined and diagnosed by core deficits in social communication and the presence of restricted and repetitive behaviors [1]. This symptom presentation is accompanied by differences in how individuals process information, including social information [2, 3]. For example, atypicalities in ASD have been observed across infants, children, and adults and at various levels of specificity, including decreased attention to social scenes compared to nonsocial (objects or geometric patterns) scenes [4–7] and reduced eye gaze directed toward social aspects of complex scenes [6, 8–15]. Although infants at risk for developing ASD may not show this global social orienting deficit [16], these findings, across development, suggest that ASD is linked with deficits in effectively globally orienting to social stimuli.

Faces are one of the most important visual cues for social functioning. In ASD, disrupted face processing has been found across a variety of domains, including differences in how individuals with ASD view faces [8, 17–21] and decreased face identification accuracy ([22] for a recent review). In addition, findings from neurotypical adults suggest that simple face-related information (e.g., identity, gender, emotional expression) is processed rapidly, and depends specifically on the initial saccades to a face [23–25]. At this point, the functional consequence of disrupted eye gaze patterns to faces on simple face-related tasks, such as face identification, remains underexplored in ASD.

### Eye gaze patterns to faces in ASD

When examining gaze patterns to faces, individuals with ASD consistently show decreased gaze to the eye region when the face is presented within social scenes [8, 17–21] ([6, 26]; but see [21]). This decrease in viewing the eyes is particularly relevant because the eyes are a highly informative area supporting face recognition [27], theory of mind [28], and joint attention [29, 30], all of which are implicated in ASD. In addition, a recent review posits “eye avoidance,” or active avoidance of the eye region, as a plausible explanation for face processing deficits in ASD [31].

However, the evidence of altered gaze patterns in ASD is mixed for studies presenting faces in isolation. Some studies have shown reduced fixations to the eyes and/or increased fixations to the mouth in ASD [32–35], but other studies have failed to report any location-specific gaze differences [36, 37]. These studies presented images of isolated faces for durations ranging from 2 to 10 s and averaged the number of fixations within regions of interest on the face (e.g., eyes, mouth). These mixed findings may be due to averaging effects across space and time that likely miss moment-to-moment differences in viewing patterns. In fact, stimulus presentation duration has been proposed as one factor that may explain these discrepant findings [38], and there is some evidence that eye gaze patterns in ASD may be selectively disrupted during the most important moments in a particular context (e.g., atypical gaze patterns when the speaker switches in a conversation) [8]. In addition, the majority of these studies utilized passive viewing tasks, which may not direct visual attention in the same way as when eye gaze is required to complete a particular task (e.g., face or emotional identification).

### Face identification in ASD

Deficits in face recognition are widely acknowledged as important in the phenotype of ASD [39, 40]. However, reviews on the empirical studies of face identification present mixed evidence related to face identification deficits in ASD [41, 42]. In general, studies suggest that individuals with ASD do not demonstrate complete face blindness [42]; however more subtle differences in face identification, as described below, are apparent [41, 42]. Studies comparing faces to other (nonsocial) objects support that these deficits are face-specific in ASD [43, 44]. In addition, consistent with the idea of the broader autism phenotype, atypicalities in face recognition extend to individuals related to those with ASD [45–47] and to individuals with subclinical levels of autistic traits [46].

Focusing specifically on simple face identification abilities, some studies find deficits and others find intact face identification in ASD [41, 42]. Memory demands seem to explain some of these discrepant findings, with higher memory demands related to worse performance [42]. However, there are several studies that measure simple face discrimination without memory demands and find worse performance in ASD [44, 48–50]. Interestingly, these studies utilize paradigms that require more fine-grained perceptual discrimination and thus might tap into more subtle deficits related to face recognition in ASD. Specifically, facial identity discrimination based on the eye region seems to be a consistent deficit in ASD [50–52]. This matches the largely consistent finding of reduced eye gaze to the eye region in ASD and the “eye avoidance” hypothesis [31]. In addition, in the one study that used brief presentation durations (40–100 ms), Wallace et al. [44] found worse performance in ASD, pointing to possible differences in the abilities of adults with ASD to quickly process faces on the same timescale as neurotypical adults.

Taken together, there still remains significant uncertainty about the extent and nature of face viewing and identification differences in ASD. One strategy to address outstanding questions is to focus on increased precision by investigating face processing at specific moments that are critical to the process of interest. If individuals with ASD, for example, are impaired in functionally important moments during the process of face identification, it may be concluded that this important identification process (and perhaps face processing more generally) is not occurring efficiently. By extension, this critical moment of processing likely impacts moment-to-moment processing in complex social interactions more broadly. The following section details previous research that indicates that the initial eye movement to a face is highly informative for the process of face identification in neurotypical adults, which provides rationale to investigate this critical moment of processing in ASD.

### Initial eye gaze patterns to faces and face identification in neurotypical adults

Although humans look at faces for long periods of time when engaging in social interactions, many face-related tasks, including face identification, happen very rapidly. In a systematic study of how quickly humans identify faces, Hsiao and Cottrell (2008) [23] showed that identification performance is at its maximum after two eye movements (i.e., saccades) on the face and that additional viewing time does not provide any additional information. In addition, they showed that the first saccade provides the most information, allowing for face identification performance well above chance level.

In a series of studies that focused primarily on the first saccade to a face, it was demonstrated that neurotypical adults have an individual-specific and highly stereotypic location of the first saccade that ranges, between individuals, from the eyebrows to the mouth [23–25, 53–55]. For example, one individual may consistently initially look between the eyes while another at the bridge of the nose. These initial eye movement patterns have been shown to be robust to different stimulus presentation settings [24, 25], programmed such that they do not change even to compensate for vision loss [53], and representative of real-world looking behaviors [55].

Results from these studies also show that face identification abilities *within an individual* vary as a function of where that individual looks on the face [24, 25, 54]. Experimentally, this has been tested by briefly showing images of faces while participants are forced to look at a specific location on the face during an identification task. On average, individuals show the best identification abilities when they are looking right below the eyes, compared to other locations along the midline of the face [24]. This matches natural looking patterns, which show that on average, individuals look just below the eye when presented an image of a face [24]. Furthermore, within an individual, performance is better when one is looking at the location that best matches his/her preferred natural initial eye movement location compared to when one is looking at other locations along the midline of the face [25, 54]. Together, these results suggest that each individual may develop his/her own specific looking pattern with an associated optimal face identification performance for him/herself.

It is plausible that the known face processing deficits associated with ASD begin early in a complex process that unfolds over time and may include several sequential processing steps. Specifically, individuals with ASD may, on average, show disruptions in their initial eye gaze patterns to faces and their consequence on face identification. Based on evidence supporting the eye avoidance hypothesis [31], one specific prediction is that individuals with ASD may initially look lower on faces compared to typically developing (TD) controls, which may or may not be associated with poorer face identification performance. Another possible result is that individuals with ASD show increased variability across trials in their initial fixations to faces, which could limit the presence of an individually specific optimal initial fixation location in this population. Because the initial moment of face processing has been shown to be critical to efficient face identification, atypicalities in the first saccade to a face may have significant impact on basic face-related tasks (e.g., face identification) and could further lead to delayed or belabored processing throughout the duration of a social interaction. In other words, if the first moment of processing is disrupted each time an individual looks at a face, it likely leads to dysfunction in not only that first moment, but also all subsequent moments of that interaction.

Finally, although this literature points to individual differences in initial eye movements in neurotypical adults, the basis for this individual variability remains unknown. An important next step includes understanding how differences in initial looks to faces may be systematically related to other differences across people. Consistent with the idea of the broader autism phenotype [56–58], it is plausible that variability across people in initial eye gaze to faces is related to individual differences in symptoms related to ASD (e.g., social communication and repetitive behaviors). Utilizing a broader autism phenotype framework, which posits that autistic traits are continuously distributed across the entire population [58, 59] with the clinical disorder existing at the upper end of that distribution, can elucidate how specific mechanisms may be disrupted across subclinical and clinical levels of autism symptomatology.

### Current Study

The purpose of this study was to characterize the initial eye movement to a face, one critical moment of face processing, and examine its impact on face identification abilities across three groups: (1) adolescents with ASD, (2) TD adolescents, and (3) neurotypical adults. This allowed us to explore our primary questions related to how this critical moment of face processing may be atypical in ASD and to explore an additional aim of how individual differences in this critical moment of processing may be related to autistic traits in the general population.

To do this, we adapted an established eye-tracking and four-alternative-forced-choice face identification paradigm, which allowed us to investigate how individuals with ASD may differ in (1) location of their initial eye movement to a face, (2) face identification accuracy for briefly presented face stimuli, and (3) the extent to which initial eye movements to faces support optimal face identification. In addition, we included a separate behavioral face identification task to better understand how possible differences in ASD in the critical initial moment of face processing extend to untimed and perceptually more complex face identification.

In addition, a group of neurotypical adults completed the main eye tracking and associated face identification behavioral task. This allowed us to best compare our results in the novel populations of adolescents with and without ASD to the existing neurotypical adult literature. By additionally collecting a widely used self-report measure of autistic traits in this group, we were also able to explore individual differences in how initial eye movements to faces and face identification on a brief timescale may be related to autistic traits in the general population, motivated by the idea of the broader autism phenotype.

## Methods

### Participants, recruitment, and screening

#### Participant characteristics

The sample included 21 adolescents (20 male) with ASD, 21 TD controls (all male), and 41 neurotypical adults (all male). All of these participants completed the main initial fixation to faces task. Of these, 13 adolescents with ASD, 20 adolescents with TD, and 39 adults completed the forced fixation task. See Additional file 1 for an explanation for the reduced sample in the forced fixation task, as well as supplemental analyses examining differences between adolescents with ASD who did and did not complete this task. Briefly, these additional analyses did not reveal any substantive differences between these subgroups and supported all main conclusions reported in the paper. Adolescent groups were matched on both age and measures of IQ. See Table 1 for detailed participant characteristics in the adolescent groups. Adult participants were 18–22 years old (mean age = 20.10).

**Table 1:** Adolescent participant characteristics

	ASD (*n* = 21)				TD (*n* = 21)		Statistical Comparison	
	Mean	(SD)	Range	Mean	(SD)	Range	*t*	*p*
Age	14.11	(1.46)	12.12–16.80	14.89	(1.79)	12.01–17.96	1.55	.13
Full Scale IQ	104.52	(11.84)	84–133	107.24	(12.15)	78–126	.73	.47
Performance IQ	105.76	(13.76)	77–132	105.38	(12.84)	77–131	.09	.93
Verbal IQ	102.62	(13.75)	71–138	107.62	(9.78)	85–122	1.36	.18
ADOS-2 Severity	6.48	(1.66)	3–9	1.10	(.30)	1–2	14.6	< .001

Note: All IQ scores are estimated from the WASI-II or abbreviated versions of the WISC-IV or WAIS-IV. ADOS-2 Severity is the calibrated severity score, which ranges from 1 to 10, with higher numbers reflecting more severe ASD symptoms [60, 61]

Adolescent participants were recruited to be 12–17 years old with FSIQ above 75. We chose this developmental period for two reasons. First, the most substantial developmental improvements in simple face identification occur by around age 11 [62–64]. Thus, we chose to limit our sample to begin after this developmental period. Second, to minimize noise in the data that may be caused by normal developmental changes in face processing mechanisms [65, 66] and to effectively characterize these behaviors in a developmental disorder such as ASD, it is important to conduct this study with participants within a limited developmental period, such as adolescence. Thus, we chose to limit our upper age range to 17 years. To ensure participants could successfully understand and complete the tasks, we limited our sample to individuals with FSIQ greater than 75.

For the adolescent sample, a priori power analyses were conducted for primary *t* test analyses, which guided determination of sample size. A sample of 20 participants per group is adequate to observe group differences of large effect sizes (Cohen’s *d* ≥ .8) with 80% power. Post-hoc Bayes factor calculations were conducted to examine the likelihood of the null versus alternative hypotheses for main group comparisons, including for the forced fixation task in the reduced sample (see Results). These analyses allowed us to quantify the evidence present in the data to determine an odds ratio of how likely these results would occur under the null versus alternative hypotheses [67]. Adult sample size was larger given primary motivations to explore individual variability and possible relationships with autistic traits in this group.

#### Recruitment and eligibility

Adult participants were recruited through the University of Rochester’s undergraduate psychology participant pool, which includes college students enrolled in psychology courses. The only additional eligibility criterion was being male.

Adolescent participants were recruited from several sources, including through IRB-approved recruitment databases at the University of Rochester and University of Rochester Medical Center, flyers posted on community boards in areas surrounding the University of Rochester, and on social media sites through the University of Rochester Medical Center. Exclusion criteria for both groups included uncorrected vision (screened over the phone and confirmed at the first visit), diagnosis of a neurological disorder or injury, or injuries affecting eye movements. TD participants were further excluded if they had received other mental health (e.g., ADHD, depression, anxiety) or learning/behavioral diagnoses or if they had a first-degree relative with an ASD. Participants recruited for the ASD group were required to have a previous clinical diagnosis of ASD.

Final decisions about eligibility were determined at the first visit. IQ was estimated via the Wechsler Abbreviated Scale of Intelligence (WASI-II [68];) or a short form of the Wechsler Intelligence Scale for Children (WISC-V [69];) or Wechsler Adult Intelligence Scale (WAIS-IV [70];) that included the same four subtests as the WASI-II. In the ASD group, diagnoses were confirmed by research-reliable administration of the Autism Diagnostic Observation Schedule-2 (ADOS-2 [71];) and the Social Communication Questionnaire (SCQ [72];), and clinical judgment by a licensed clinical psychologist. Administration of the Autism Diagnostic Interview-Revised (ADI-R [73];) was additionally used in the majority of participants. Fourteen participants had previously received the ADI-R through other studies in our lab, and one additional participant was administered the ADI-R to resolve diagnostic ambiguity. In the TD group, diagnoses were ruled out with a combination of the ADOS-2 and SCQ, plus clinical judgment. The ADOS-2 calibrated severity score [60, 61] was calculated as an index of ASD symptom severity. Scores on this measure range from 1 to 10 with higher scores reflecting greater levels of ASD severity (see Table 1). All participants completed a vision screening via Snellen eye chart to confirm visual acuity of 20/40 or better.

All procedures were approved by University the of Rochester’s Research Subjects Review Board. Adult participants provided informed consent and received extra credit in their psychology courses as compensation. For adolescents, caregivers provided written informed consent for their child’s participation, and adolescents provided written or verbal assent, depending on their age. Adolescent participants received monetary compensation for participation.

### Experimental design

Following a separate diagnostic visit (detailed above), adolescent participants completed all experimental study procedures in a single visit, lasting approximately 2.5 h. At this visit, they completed all experimental tasks in a fixed order: (1) free viewing of briefly presented faces (task training/face familiarization followed by face identification phases), (2) forced fixation of briefly presented faces, (3) control task (free viewing of snowflakes; see Additional file 1), and (4) the Dartmouth Face Perception Test (DFPT; all tasks detailed below). An interactive visual schedule was used for all adolescents to help track progress and maintain engagement.

Adult participants completed two experimental sessions, each lasting approximately 2 h. During visit 1, participants completed a vision screening via Snellen eye chart, the free viewing task, and the Autism Quotient (AQ) questionnaire. On visit 2, participants completed the forced fixation task. Of note, adult participants completed additional trials of both experimental tasks to aid in paradigm development; however, only trials completed across all three participant groups were used in analyses presented here. See Additional file 1 for an explanation of paradigm development and additional analyses using all trials collected in the adult sample.

#### First look face tasks

For all first look face tasks, participants were comfortably seated 135 cm from a projection screen (enforced via chin rest). Stimuli, created in MATLAB and the Psychophysics Toolbox, were shown on a natively linear DLP projector (DepthQ WXGA 360; 1280 × 720 resolution; 120-Hz frame rate). Viewing was binocular. The ambient and background illumination were 1.8 and 113.7 cd/m^2^. Eye gaze was tracked using an SR Research EyeLink 1000 Tower Mount eye tracker. Prior to beginning each task block, a 9-point calibration and validation procedure ensured a mean error of no more than 0.5° of visual angle.

Stimulus parameters and task design (described below) were similar to a previously used lab paradigm [24, 25] with one main modification; we used four, instead of ten, face stimuli for the face tasks. These four stimuli were selected from the set of ten faces used in the previous studies of neurotypical adults. Specifically, stimuli were four gray scale, frontal photographs of male faces, cropped to remove background, hair, and clothing, and scaled to achieve equal proportions across the faces. The size of the face stimuli was 18.5° × 18.5° visual angle. We used only four faces to simplify and shorten the task for our participants. An ideal observer analysis [24] confirmed that the stimulus-driven optimal viewing points did not differ in our smaller stimulus set compared to the full ten face stimulus set used previously. Additional modifications were made using a data-driven paradigm development approach (see Additional file 1).

##### Free viewing of briefly presented faces

**Fig. 1:**
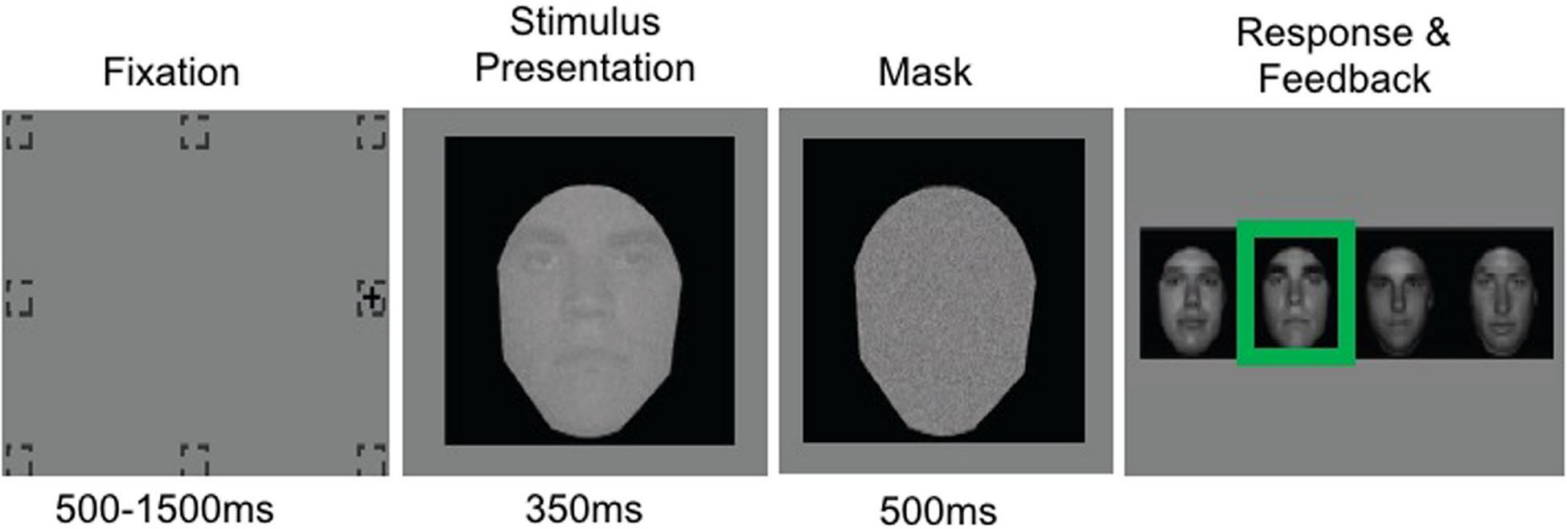
Design for face identification free viewing task. Dashed boxes represent possible fixation locations. Stimuli presented at 15% contrast during stimulus presentation stage. Green box represents correct response. Note: all-black backgrounds are used here to aid visibility of stimulus but do not appear during experiment

The task consisted of two phases: a task training/face familiarization phase followed by the face identification phase. Across both phases, each trial included four stages: fixation, stimulus presentation, mask, and response (Fig. 1). During fixation, a fixation cross appeared in one of eight locations around the periphery of the screen. Fixation locations were in the periphery to eliminate the potential confound of initial fixation bias [23, 74], and the fixation period varied across trials (500–1000 ms in adolescents and 500–1500 ms in adults) to prevent learning when the stimulus will reliably appear. Then, the face stimulus was presented and participants were able to freely move their eyes. Following stimulus presentation, a mask covered the stimulus and then four pictures were presented. The participant used a response keypad to indicate which of the faces was previously shown. Auditory and visual feedback regarding accuracy was given after each trial.

If the participant broke fixation (gaze drifts > 1.5° of visual angle from center of fixation cross) during the fixation stage, the trial did not proceed to stimulus presentation, and fixation was instead repeated until successful. However, for some adolescent participants (6 ASD and 2 TD), this fixation criteria led to repeated fixation failures due to involuntary eye movements. For these participants, we confirmed that calibration was acceptable and then increased the tolerance of fixation to 2° of visual angle, which allowed successful continuation with the task, except for one ASD participant whose tolerance had to be increased to 3.3° of visual angle. Excluding these participants from the analysis did not affect any of the results.

The purpose of the task training/face familiarization phase (3 blocks in adolescents and 4 blocks in neurotypical adults; 60 trials per block) was to train participants to maintain appropriate fixation and to learn the faces so that they could successfully discriminate between them. As such, we used a procedure that allowed for gradual exposure to stimulus parameters and presentation times that were later used in the face identification condition (see below). Block 1 included high contrast (30%) stimuli and long presentation duration (1.5 s), block 2 included low contrast (15%) stimuli and short presentation duration (350 ms), and block 3 included low contrast stimuli and short presentation duration (matching the stimulus parameters of the 4 experimental blocks that follow).

The face identification phase (4 blocks of 60 trials each) provided the main data of interest. In this condition, participants naturally and quickly look at a face. These trials included face stimuli (15% contrast) presented for 350 ms. This stimulus presentation time was selected to allow for a single saccade; a single saccade takes ~ 200 ms to initiate and lasts 20–250 ms. Pilot testing suggested that 15% contrast was optimal for participants to be able to perceive the faces, but keeps identification accuracy below ceiling (100% correct identification).

##### Forced fixation of briefly presented faces

**Fig. 2:**
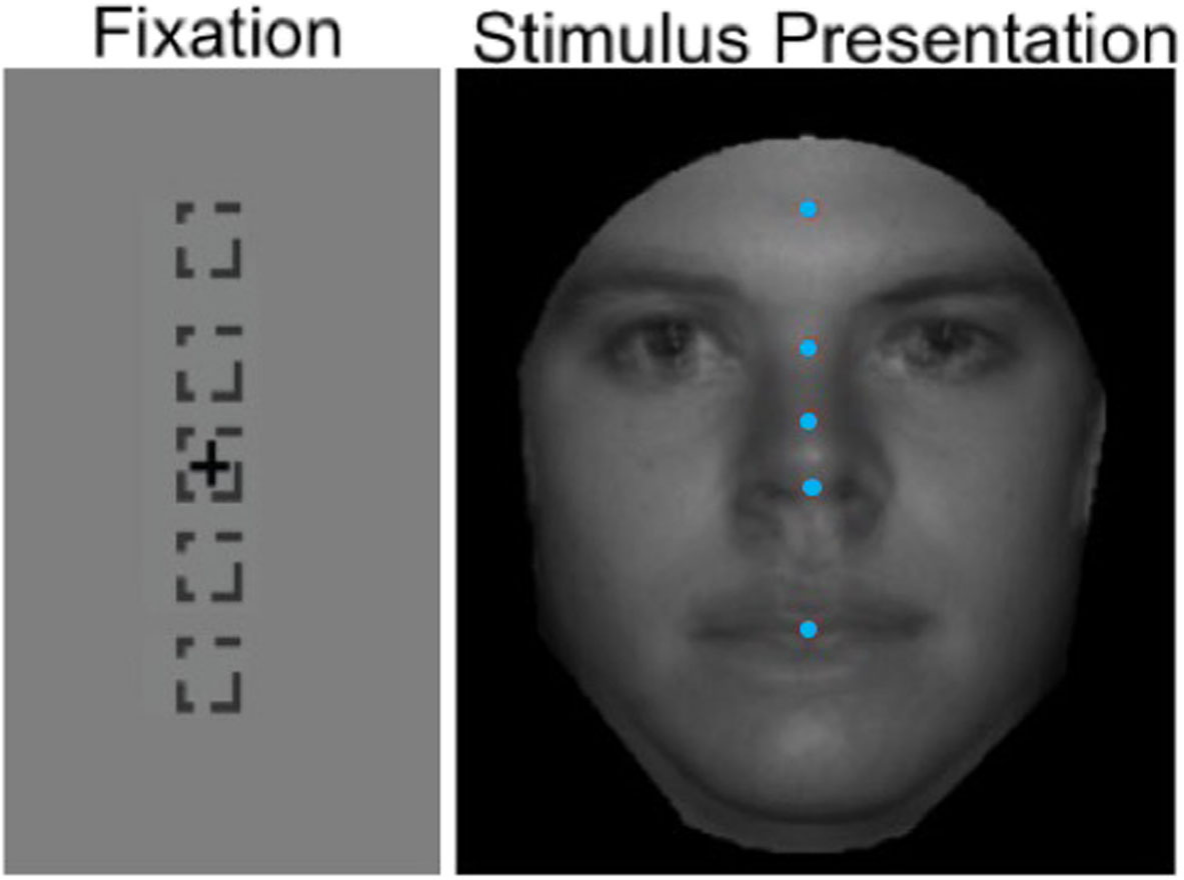
Fixation and stimulus presentation stages for forced fixation task. Four face stimuli are superimposed on top of each other for illustration, with fixation positions (blue dots) indicated along the midline of the face. A fixation cross (and not blue dots) for the specific fixation location for each trial remained on the screen during stimulus presentation

The forced fixation task was similar to the free viewing task, with the main difference being that participants were not able to freely view the face stimulus but rather were instructed to look at a specific location on the face. To “force” fixation, participants were required to fixate at a location that corresponded to a specific point on the face so that their eyes were fixated on that specific location when the face stimulus appeared (Fig. 2). In this way, we were able to experimentally manipulate the visual information on the face that was perceived on each trial. Participants completed 7, 60-trial blocks with fixation location randomly selected to be one of five locations along the midline of the face: forehead, between the eyes, middle nose, tip of nose, mouth (84 trials total per fixation location). The same fixation break criterion outlined in the free viewing task was used; however, it now applied to both the fixation and stimulus presentation stages to ensure gaze to the experimentally manipulated location on the face.

#### Dartmouth Face Perception Test (DFPT)

All adolescent participants also completed the DFPT [75], a face identification task developed as a child-appropriate version of the Cambridge Face Perception Test [76]. Seven additional participants (5 ASD and 2 TD), characterized by and eligible based on the same criteria as described previously, completed only the DFPT, thus increasing the sample size to 26 ASD and 23 TD for this task. These additional participants were unable to complete the main experimental tasks due to eye-tracking calibration difficulties caused by the glasses they wore to correct for visual impairments (*n* = 5) or participants choosing to discontinue participation due to low frustration tolerance (1 ASD) or a recent wrist injury (1 TD). The DFPT is a three-alternative forced-choice task that consists of 40 trials (approximately 10 m) and specifically measures perceptual aspects of face identification. Each trial consisted of a target face presented facing 30° to the viewer’s left, with three frontal-view faces as response choices presented below the target. The participants’ task was to choose which of the three faces was the same identity as the target face. Stimuli included photographs of eight male and female child faces from the Dartmouth Database of Children’s Faces [77]. All faces had neutral expressions, were cropped to remove hair and ears, and converted to gray scale. From these target faces, choice faces were created by morphing the target face with a same-gender distractor face. Choice faces varied along a morph continuum from 10 to 90% target identity.

The task differed from the face identification component of our main experimental task (first look face task) in several ways. First, the target face was presented for an unlimited duration, which removed the quick processing demand and allowed a more thorough exploration of the faces. Second, the choice faces were morphed versions of the target face rather than being completely different identities. Finally, the target and choice faces were presented at different perspectives; the target faced 30° to the viewer’s left and the choices are presented at frontal-view. Thus, this is a measure of perspective-invariant and time-unlimited face discrimination abilities. This allowed us to better isolate the face identification differences in individuals with ASD and to test for relationships between various face identification measures.

#### Autism Quotient (AQ)

The AQ, a 50-item self-report inventory of autistic traits, was collected in our neurotypical adult sample. The AQ can be used to sensitively measure autistic traits in the general (non-clinical) population [57], supporting the idea that these traits exist on a spectrum extending into subclinical and non-clinical samples. Participants read a series of statements (e.g., “I enjoy social chit-chat,” “I know how to tell if someone listening to me is getting bored”) and respond on a 4-point Likert scale from “definitely disagree” to “definitely agree” based on the degree to which each statement applies to them. Although responses are on a 4-point scale, scoring is binary whereby each item is either scored as a “1” or “0,” collapsed across “definitely agree”/“agree” and “definitely disagree”/“disagree.” Possible scores range from 0 to 50, with higher scores indicating the presence of more autistic traits.

### Data analysis

#### Characterization of initial eye movements to faces

##### Free viewing of briefly presented faces

Experimental trials from the four blocks of the face identification condition, where stimuli were briefly presented (350 ms) at low contrast (15%), were used in this analysis. For each trial, we calculated the landing location (in degrees of visual angle below the eyes) that followed the first registered saccade during the stimulus presentation stage. Saccades were classified as events where eye velocity and acceleration exceed 22°/s and 4000°/s^2^, respectively; these settings adequately capture saccades in adults as well as ASD and TD adolescents [78]. Only trials in which the first registered saccade landed somewhere on the face stimulus were included in analyses. There were more excluded trials in the ASD group compared to the TD group, *t*(40) = 3.0, *p* = .005. To test whether this difference affected our findings, we conducted supplemental analyses and showed that our main findings remain nearly identical if we exclude ASD participants who had high numbers of excluded trials (see Additional file 1).

Two variables were calculated for each participant across trials: (1) average location and (2) standard deviation (SD) of the initial eye movement to faces. Percent correct identification was calculated from the participants’ responses. Comparisons between our three participant groups were made using ANOVA with post-hoc LSD tests, as appropriate.

##### Forced fixation of briefly presented faces

Percent correct identification was calculated at each forced fixation location. A 3 (participant group) × 5 (forced fixation location) mixed-model ANOVA was conducted to assess the accuracy pattern (percent correct identification) as a function of fixation location and to assess how that pattern may differ in neurotypical adults, adolescents with TD, and adolescents with ASD. Follow-up *t* tests were conducted as appropriate.

##### Free viewing related to forced fixation

Next, we examined how participants’ preferred initial eye movement locations were related to their pattern of performance on the forced fixation condition, consistent with analysis approaches in other studies using this paradigm [25, 55]. Using accuracy measures at a high (eyes) and low (mouth) forced fixation location, we calculated a difference score to represent the degree of differentiation in performance when forced to look higher versus lower on the face. Positive values represent better performance at the lower compared to the higher location, whereas negative values represent better performance at the higher compared to the lower location. Correlations, separate by group and combined across groups, were conducted between the forced fixation difference score (performance at mouth minus performance at eyes) and each individual’s average first fixation location from the free viewing condition. This allowed us to assess the degree to which natural first fixation locations influenced differences in performance when participants were forced to look at higher and lower face locations.

#### Dartmouth Face Perception Test (DFPT)

Only adolescents with and without ASD completed this task given that it was designed for use in children. Two variables were of interest from the DFPT: percent correct and mean reaction time. Although participants were not instructed to respond as quickly as they could, reaction time was collected on each trial as a measure of how quickly individuals naturally perform this face identification task. Mean reaction time was calculated by taking the average reaction time across trials after removal of outliers (> 2SD from the mean). Groups were compared using *t* tests. Pearson correlations between accuracy on the DFPT and face identification accuracy during the free viewing condition of the first look face task were also conducted in each group separately to determine relationships across the two face identification tasks.

#### Associations with autistic traits

Several correlations were explored to examine relationships with autistic traits as measured by the AQ questionnaire in our adult sample. First, we aimed to understand if the AQ total score was related to any natural initial eye movement variables (mean and standard error in the vertical dimension) and face identification accuracy from the free viewing condition. Next, we examined whether AQ was related to any variables from the forced fixation condition (peak performance, face location corresponding to peak performance, difference score between performance at mouth and forehead) and/or any difference scores between the free viewing and forced fixation conditions (free viewing and peak forced fixation performance, free viewing look location and peak look location from forced fixation). Pearson correlations were conducted to examine each of these relationships.

## Results

### Characterization of initial eye movements to faces in adolescents with and without ASD and neurotypical adults

To preview the results related to our main aim investigating possible differences in ASD, adolescents with ASD and TD showed strikingly similar patterns of behavior related to initial eye gaze to faces and face identification performance for briefly presented faces across both the free viewing and forced fixation conditions.

#### Free viewing of briefly presented faces

**Fig. 3:**
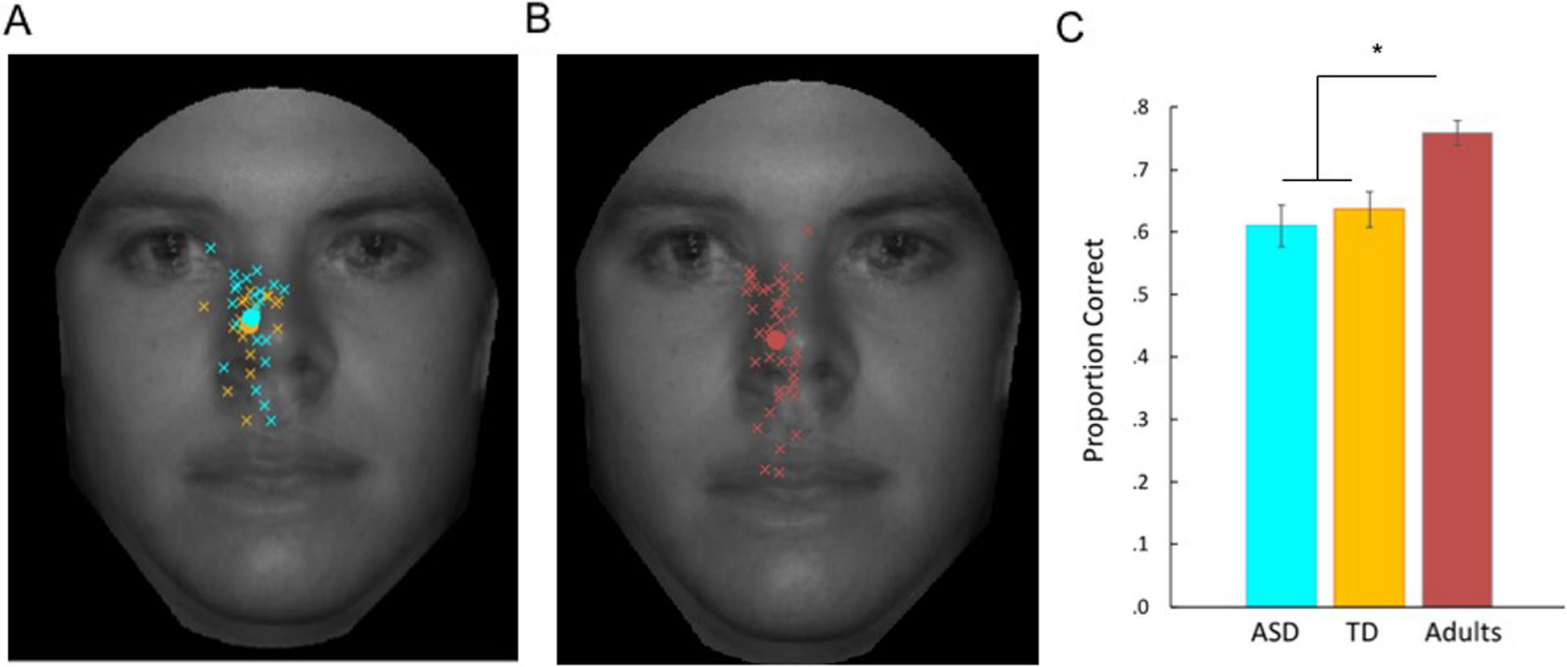
Results from face identification condition of free viewing task. **a**, **b** Average initial eye movement locations for each participant (ASD: cyan, TD: orange, adults: red). Average initial eye movement location across participants (filled in circles). Four faces are superimposed on top of each other for illustration. **c** Proportion correct identification

Across all three groups, individuals, on average, made their initial eye movement to a face between the eyes and nose (ASD = 1.54° below the eyes; TD = 1.75° below the eyes; adults = 2.07° below the eyes; Fig. 3a, b). Standard deviation of the first look location in the vertical dimension was also calculated in the three groups (ASD = 1.83, TD = 1.78, adults = 1.63). There were no group differences in the average location of the initial eye movement, *F*(2,82) = 1.21, *p* = .30, or in the variability (SD) of landing location of the initial eye movement across trials, *F*(2,82) = 1.40, *p* = .25, in the vertical dimension. This suggests that initial eye gaze to faces, which is critical to rapid face identification, is similar across neurotypical adults and adolescents with and without ASD.

Performance on the face identification task did differ between groups, *F*(2,82) = 10.41, *p* < .001. Follow-up LSD tests indicated that adults performed better than both TD (*p* = .001) and ASD (*p* < .001) adolescents, but that performance did not differ between the ASD and TD adolescents (*p* = .53; Fig. 3c). See Additional file 1 for results from a control task using non-face stimuli.

Because of the unexpected lack of group differences between our ASD and TD adolescent groups, we conducted a direct comparison between these two groups. Independent samples *t* tests confirmed that there were no group differences in the average location of the initial eye movement, *t*(40) = .61, *p* = .54, *d* = .19, or in the variability (SD) of landing location of the initial eye movement across trials, *t*(40) = − .32, *p* = .75, *d* = .10, in the vertical dimension. In addition, performance on the face identification task did not differ between groups, *t*(40) = .60, *p* = .55, *d* = .19. Results were similar when only correct trials were compared between groups: average location of initial eye movement, *t*(40) = .34, *p* = .74, *d* = .10; variability (SD) of landing location of initial eye movements across correct trials, *t*(40) = − .90, *p* = .37, *d* = .28. Although differences in initial fixation location were observed across the eight starting fixation locations, *F*(7,287) = 74.24, *p* < .001, and accuracy differed as a function of which one of the four faces was shown, *F*(3,120) = 65.1, *p* < .001 these patterns were very similar across the two groups and no effects of group or interactions were observed (all *p* > .5). This supports our main conclusion that the initial look to faces is remarkably similar across ASD and TD groups.

Furthermore, post-hoc Bayes factor analyses [79, 80] were conducted to more conclusively determine the likelihood of these null effects (as opposed to a lack of power to detect statistically significant differences). Bayes factor values less than 0.33 are indicative of evidence in favor of the null hypothesis, with values less than 0.10 strongly indicative. Values greater than 3.0 are indicative of evidence in favor of the alternative hypothesis. Values between 0.33 and 3.0 remain inconclusive based on the data [81, 82]. Based on the theoretical possibility that our effects could be in either direction, we used a mean of *p*(population value|theory) of 0 and a 2-tailed distribution. The standard deviation of *p*(population value|theory) is defined as the maximum plausible effect and was set to the difference score between the highest and lowest individual values from our sample [80]. Across these three variables (mean location of initial eye movement, variability of initial eye movement location across trials, and percent correct identification), the Bayes factors ranged from 0.06 to 0.09. This confirms that our data strongly support the null hypotheses whereby there are no differences between adolescents with and without ASD in initial eye movements to faces or rapid face identification abilities.

In addition to testing the main variables of interest from the free viewing task, we also compared our three groups on average landing location of the initial eye movement to faces and variability across trials in the horizontal dimension; we found no group differences (average location of initial eye movement: *F*(2,82) = .85, *p* = .43, SD of initial eye movement locations across trials: *F*(2,82) = .75, *p* = .48). Together, these data suggest that the initial eye movement to a face and one corresponding functional consequence of that first moment of processing (face identification) is intact in ASD.

#### Forced fixation of briefly presented faces

**Fig. 4:**
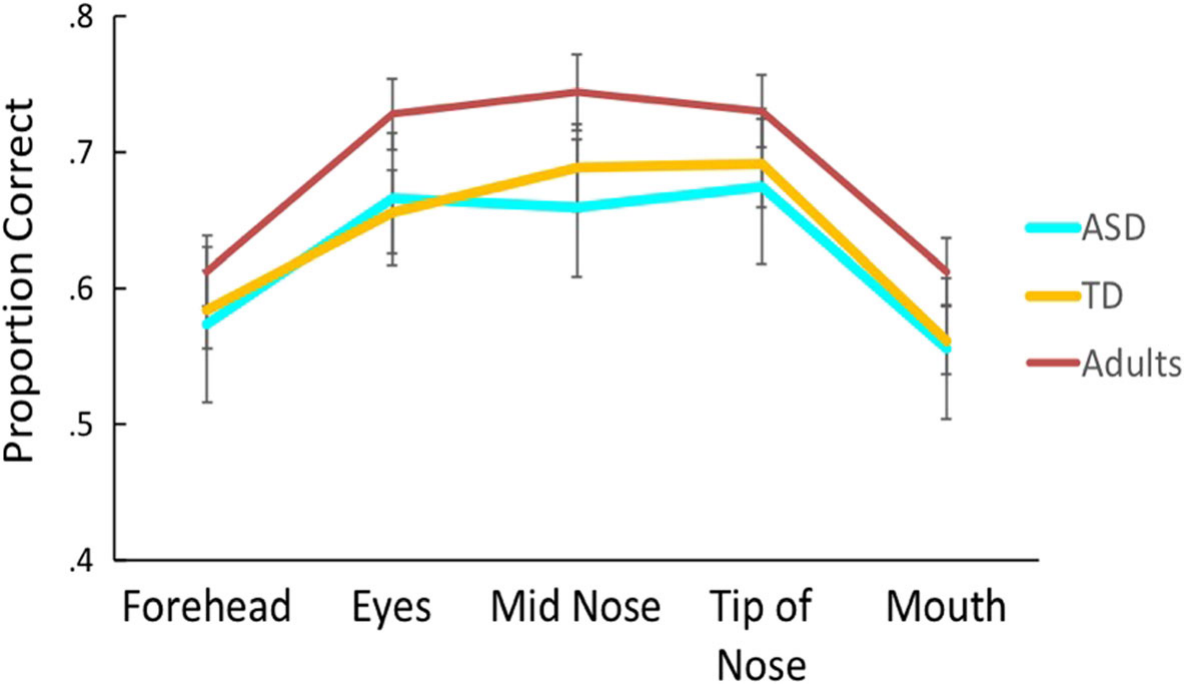
Results from forced fixation task. Across all groups, proportion correct face identification varied as a function of where participants were “forced” to look at the face. There were no differences in this pattern between the three groups

A mixed-model ANOVA revealed a significant effect of fixation location on face identification accuracy, *F*(4,276) = 51.70, *p* < .001, ${\eta}_p^2$ = .43. A trend analysis yielded no linear effect, *F*(1,69) = .39, *p* = .54, ${\eta}_p^2$ = .006, and a significant quadratic effect, *F*(1,69) = 153.31, *p* < .001, ${\eta}_p^2$ = .69. There was no significant effect of group, *F*(2,69) = 1.02, *p* = .37, ${\eta}_p^2$ = .03, nor a group by fixation location interaction, *F*(8,276) = .63, *p* = .75, ${\eta}_p^2$ = .02 (Fig. 4). Performance was 10–14% lower at the two extreme locations (forehead and mouth) compared to the three central locations (eyes, middle of nose, tip of nose) across all groups. Follow-up paired *t* tests, collapsed across groups, confirmed these differences, showing that performance at the forehead location was significantly lower than performance at the eyes, middle of nose, and tip of nose locations. Similarly, performance at the mouth location was significantly lower than performance at the eyes, middle of nose, and tip of nose locations (all *p* < .001). Differences were not observed between eyes and tip of nose, eyes and middle of nose, middle of nose and tip of nose, or forehead and mouth (all *p* = .06–.71). Thus, across neurotypical adults and adolescents both with and without ASD, face identification accuracy varies as a function of these locations whereby performance is maximized when looking toward the center of the face and worsens at both high (forehead) and low (mouth) extremes.

#### Free viewing related to forced fixation

**Fig. 5:**
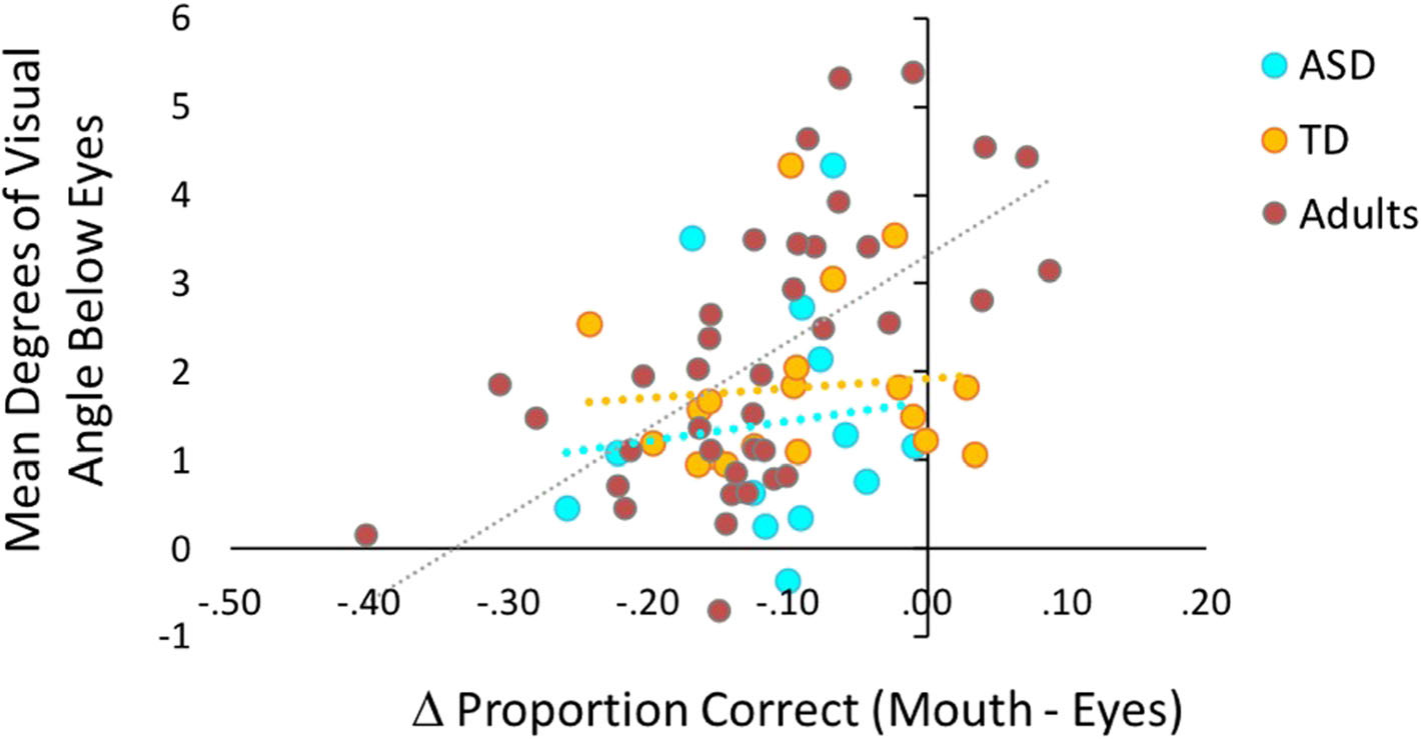
Correlation between forced fixation and free viewing tasks. In neurotypical adults, average initial eye movement location in the free viewing task (measured in degrees below the eyes) was positively related to the difference in proportion correct at the mouth and eyes locations as measured by the forced fixation task. This relationship was not observed in either of the adolescent groups

Previous literature suggests that in adults, one’s preferred initial eye movement location is related to his/her pattern of performance on the forced fixation condition, a finding that suggests individuals naturally maximize their face identification abilities when freely viewing faces. We explored whether this extended to our neurotypical adult sample and to adolescents both with and without ASD. Correlations between each individual’s average first fixation location from the free viewing condition and the difference in their performance (face identification accuracy) at high (eyes) and low (mouth) forced fixation locations were tested in each group separately. We observed the expected relationship in our adult sample, *r*(37) = .60, *p* < .001, whereby individuals who naturally look higher on the face perform better when forced to look higher compared to lower on the face and individuals who naturally look lower on the face perform better when forced to look lower compared to higher on the face. However, in adolescents, there were no significant associations (ASD: *r*(11) = .12, *p* = .71; TD: *r*(18) = .14, *p* = .56; combined adolescents: *r*(31) = .12, *p* = .49; Fig. 5). See Additional file 1 for supplemental analyses further exploring this effect across all three participant groups.

A post-hoc Bayes factor analysis was conducted on the combined adolescent sample using the Fisher z transformed correlation coefficient from our adult sample as the standard deviation of p(population value|theory). Because the theoretical prediction for this relationship is in a single direction, a one-tailed distribution was used. The calculated Bayes factor (*B* = .45) falls in the inconclusive range, suggesting that our adolescent data may not have been sensitive enough to detect this relationship.

### Dartmouth Face Perception Test (DFPT) in adolescents with and without ASD

**Fig. 6:**
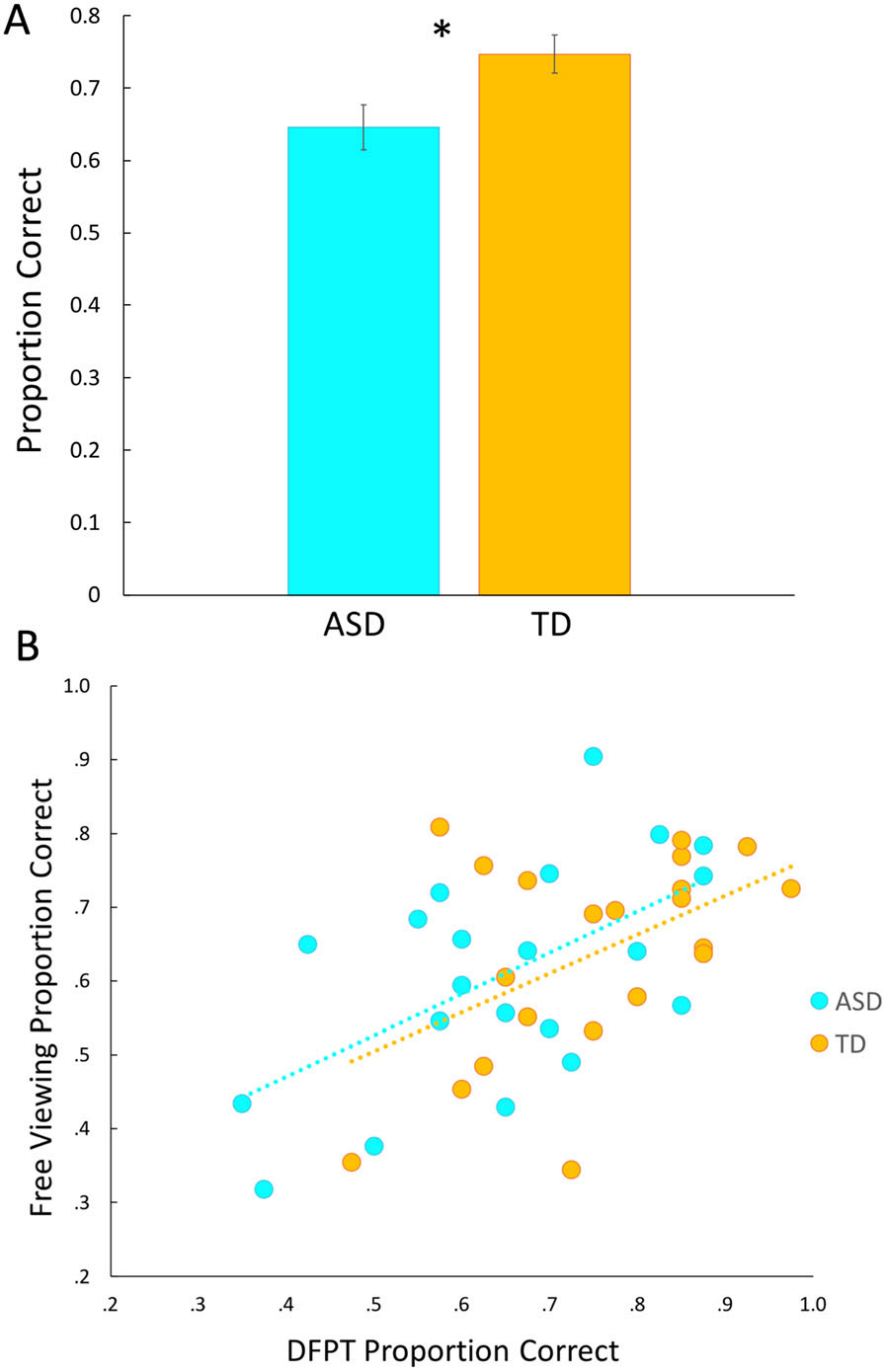
Performance on the Dartmouth Face Perception Test (DFPT). **a** ASD showed significantly worse face identification performance compared to controls on the DFPT. **b** Performance on the first look face identification task is correlated with performance on the DFPT, in both ASD and TD. Note: Statistical analyses were conducted using standardized scores but figures are plotted using proportion correct for ease of interpretation

Although there were no differences between adolescents with and without ASD in the main experimental task, group differences were revealed on the DFPT. Specifically, adolescents with ASD performed significantly worse on this face identification task compared to TD controls (Mean ASD = 64% correct; Mean TD = 75% correct; *t*(40) = 2.56, *p* = .01, *d* = .79; Fig. 6a). Adolescents with ASD also demonstrated slower reaction times compared to TD adolescents, though this did not reach statistical significance, *t*(40) = − 1.22, *p* = .23, *d* = .38. All results remained essentially identical after adding the 7 participants who completed the DFPT but not the full experimental paradigm; DFPT accuracy: *t*(47) = 2.45, *p* = .02, *d* = .70; reaction time: *t*(47) = − 1.40, *p* = .17, *d* = .40. Thus, adolescents with ASD appear to have deficits in this perceptual face identification task that targets time-unlimited face identification abilities for more perceptually complex stimuli.

### Follow-up methods and analyses

Our divergent findings whereby the first look face identification task revealed similar performance across adolescent groups while the DFPT revealed worse performance in ASD are in line with the mixed results in the current literature and highlight possible differences in ASD in specific aspects of face identification. However, given that both of our tasks were simple forced-choice face identification tasks using gray scale photographs of faces with surrounding stimuli (e.g., hair, ears, neck) removed, we would expect that these tasks rely, at least in part, on shared mechanisms. To gain an understanding of which aspects of face processing may be *different* in ASD, we must first establish whether both of these tasks are measuring the broader process of face identification. To do that, we used Pearson correlations to explore relationships between face identification performance on the free viewing task and DFPT. Proportion correct from each task was standardized to account for differences in chance levels across the two tasks (25% for free viewing task and 33.3% for DFPT) and then these *Z*-scores (calculated using group means for each task as the reference) were entered into correlations. Face identification performance across these two tasks was correlated in ASD (*r*(19) = .67, *p* = .001), TD (*r*(19) = .49, *p* = .02), and the combined sample (*r*(40) = .58, *p* < .001; Fig. 6b), suggesting that these two tasks rely in part on similar processes or abilities. However, the group difference on the DFPT persisted even when controlling for performance on the free viewing task, *F*(1,39) = 7.22, *p* = .01, ${\eta}_p^2$ = .16, highlighting that individuals with ASD have particular deficits in the processing abilities that are unique to the DFPT.

#### Associations with autistic traits in adult sample

**Fig. 7:**
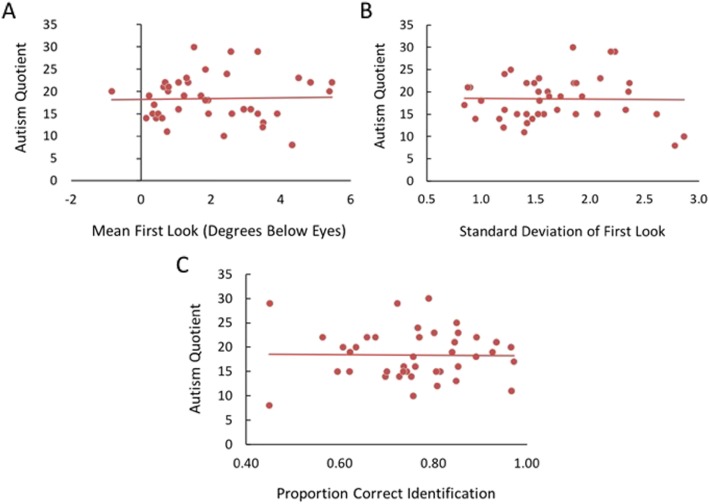
Relationships between autistic traits and free viewing task in neurotypical adults. There were no relationships between autistic traits as measured by the Autism Quotient and measures from the free viewing task: **a** Average initial eye movement location, **b** variability (SD) of initial eye movement location across trials, and **c** proportion correct face identification for briefly presented faces

The range of AQ scores in our adult sample was 8–30 with a mean of 18.4. This is consistent with the expected range and mean for this measure in a neurotypical sample [58]. Consistent with the lack of group differences on the first look face tasks between adolescents with and without ASD, there were no relationships between initial eye movement variables nor face identification accuracy with AQ scores (all *p* > .86; Fig. 7). Similarly, AQ was not related to any variables from the forced fixation condition (peak performance, face location corresponding to peak performance, difference score between performance at mouth and eyes; all *p* > .41) nor any difference scores between the free viewing and forced fixation conditions (free viewing and peak forced fixation performance, free viewing look location and peak look location from forced fixation) (all *p* > .16).

#### Exploratory analysis of relationships between the initial eye movement to faces and age

Taking advantage of the fact that the ages of the participants ranged from 12 to 17 years in our adolescent sample and 18 to 22 years in our adult sample, we explored possible developmental effects related to the initial eye movement to faces and face identification for briefly presented face stimuli.

**Fig. 8:**
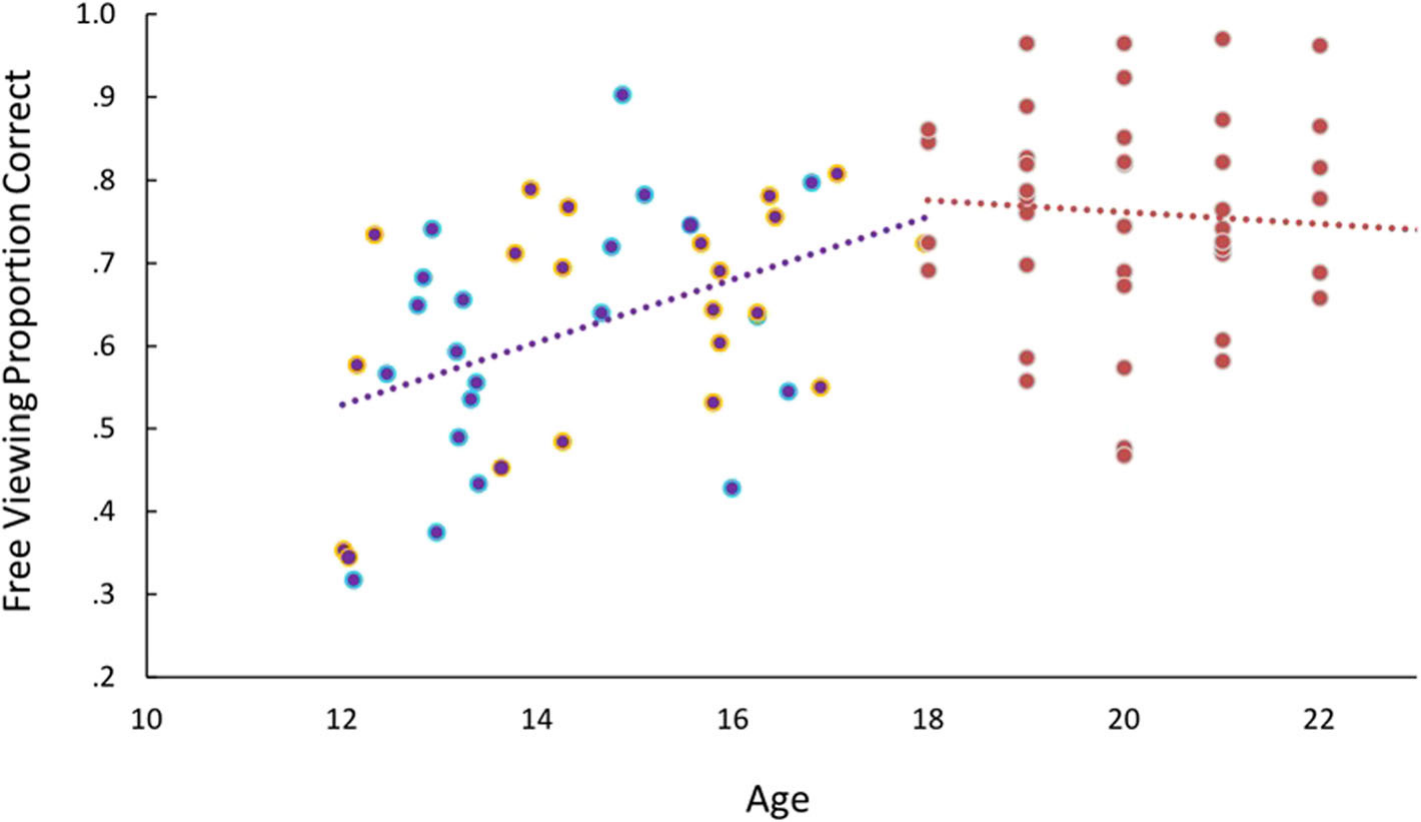
Correlation between age and face identification across adolescent and adult participants. Age is positively correlated with face identification performance on the first look task. This is true across the combined adolescent (purple; ASD outlined in blue, TD outlined in orange) and adult (red) samples and within the adolescent sample only

Pearson correlations, corrected for multiple comparisons, were conducted in our full sample (*n* = 83) between age and the following variables of interest from the first look face tasks: free viewing percent correct identification, average location of the initial eye movement, variability (SD) of the location of the initial eye movement, forced fixation accuracy difference score between low (mouth) and high (eyes) locations. After Bonferroni correction for multiple comparisons (*p* < .013 considered significant), age was significantly associated only with face identification performance, *r*(81) = .51, *p* < .001, whereby performance improved with age. This overall relationship was driven by a significant correlation between age and performance in the adolescent sample, *r*(40) = .44, *p* = .003 (Fig. 8). This relationship was not apparent in the adult sample, *r*(39) = .02, *p* = .92. In contrast, age was not significantly related to average location of the initial eye movement, *r*(81) = .12, *p* = .27, variability of the location of the initial eye movement, *r*(81) = − .20, *p* = .08, or the forced fixation accuracy difference score between low (mouth) and high (eyes) locations, *r(*70) = .05, *p* = .68.

We further explored age-related changes in the optimization of face identification abilities when naturally freely viewing faces. To do this, we divided the adolescent sample (combined across ASD and TD participants) into younger (12–14 years old; *n* = 18) and older (15–17; *n* = 15) groups. Then, Pearson correlations were conducted between each individual’s average first fixation location from the free viewing condition and the difference in their face identification accuracy at high (eyes) and low (mouth) forced fixation locations, separately in these two groups of adolescents and in the group of adults. Younger adolescents did not show any relationship between these two variables, *r*(16) = –.06, *p* = .81, whereas older adolescents began to show a positive relationship between these two variables, *r*(13) = .39, *p* = .16. Although this relationship is not yet significant in these older adolescents, like it is in adults (*r*(37) = .61, *p* < .001), this analysis provides initial evidence that maximization of face identification abilities when naturally freely viewing faces may continue to develop through late adolescence and even into early adulthood. In other words, optimal points of initial fixations to faces may be a relatively late or long developmental process.

## Discussion

Our study demonstrates that adolescents with and without ASD show remarkably similar patterns in their initial eye gaze to faces and their ability to rapidly identify faces. In addition, both groups showed similar patterns of identification performance when guided to look at specific locations of the face. Individual differences in this identification pattern were not related to preferred initial look location in these groups of adolescents, a finding that is different from that found in our adult participants and previous studies of neurotypical adults. Despite extraordinary overlap across groups in the initial eye movement to faces and face identification performance for briefly presented faces on the first look face tasks, adolescents with ASD performed significantly worse on a different, more traditional, measure of face identification.

### Initial eye gaze to faces and resulting rapid face identification appear intact in ASD

Our findings of no group differences in initial eye gaze to faces and its functional consequence on face identification performance suggest that this first moment of face processing is largely intact in ASD, at least in adolescence. Specifically, the free viewing condition measured *natural* initial eye movements to faces and corresponding face identification abilities. Adolescents with well-characterized ASD, on average, showed a similar location of the initial eye movement compared to age- and IQ-matched TD controls. Both groups showed a similar range of preferred initial eye movement locations, ranging from the eyes to above the mouth. In addition, individuals across both groups showed similar variability in the locations of the initial eye movements across trials. Furthermore, the pattern of performance on the forced fixation condition showed considerable overlap across groups in face identification performance at different locations along the midline of the face. Together, these findings all converge to suggest that the initial moment of processing in the complex temporal hierarchy of face processing is intact in ASD, at least at the developmental stage of adolescence. These results were further supported by a lack of relationship between any of these first look behaviors and self-reported autistic traits in our adult participants. This suggests that, in addition to intact initial eye gaze and face identification for briefly presented faces in adolescents with clinically significant ASD, these behaviors are not associated with subclinical symptoms in a neurotypical adult sample.

Our findings that individuals with ASD are unimpaired in this initial moment of face processing are important and encouraging. In addition to understanding where breakdowns in processing pipelines occur in ASD (or other clinical populations), it is equally important to identify aspects of processing that are intact. The initial eye movement to a face is considered a highly stereotyped behavior [25] that is resistant to change even through repeated practice and exposure [53]. Although this idea initially motivated our hypotheses that initial eye movements to faces and corresponding face identification abilities may be impaired in ASD and may contribute to the social communication challenges in this population, our findings allow us to identify and target other specific aspects of face processing that may be more malleable and thus more fruitful targets for intervention.

The lack of group differences in initial eye gaze to faces suggests that adolescents with ASD are able to plan and then subsequently execute a saccade to a specific location on a face [55, 83]. However, whether this initial look is automatic or a result of executive functions remains an open question for future investigations. Currently, a model has been proposed specifically related to the first look to a face [55], which posits that face detection and recognition are fundamentally different, but complementary, processes. This model includes five steps: detect, prioritize, select, saccade, and recognize. Although the first four steps involve significant “planning,” it is conceptualized as a reflexive process that occurs without conscious control. It will be important for this model to be tested more completely and subsequently applied to special populations, such as autism, to best understand the mechanisms for viewing and processing faces. Similar face identification performance across the ASD and TD groups further suggests that specialized mechanisms for face recognition are also intact. Thus, both the face detection and face recognition processes appear intact in ASD, at least as they relate to the rapid face processing that supports basic perceptual tasks such as face identification.

The sparing of this process is in line with a body of research that highlights largely intact face recognition in ASD for static images [41, 42]. Our finding of intact detection and rapid recognition extends this conclusion to one specific and informative moment of processing, the initial eye movement to a face. In other words, by comprehensively characterizing the initial eye movement to a face and its functional consequence of face identification, we can rule out the possibility that social information processing deficits begin at that initial moment. Instead, it is likely that processing deficits exist downstream of the initial eye movement and that those deficits contribute to the hallmark social difficulties in ASD. Alternatively, one possibility is that this highly controlled lab-based task is not representative of real-world looking behaviors. However, it has been shown that preferred initial eye movement locations to static images in the lab are strongly correlated with real-world gaze patterns in neurotypical adults as assessed using mobile eye trackers with individuals walking around a college campus [55]. Nonetheless, given that individuals with ASD show more reliable differences in eye gaze patterns when viewing dynamic compared to static faces [38], it is possible that a difference in initial eye movements would emerge with more dynamic or real-life stimuli. An important next step is to evaluate initial eye movements in a real-world context in ASD and determine whether the strong association between initial eye movements measured in the lab and in a more naturalistic context extends to the ASD population.

The sparing of this process is also consistent with ideas of intact or even enhanced low-level perceptual functioning in ASD, such as pitch discrimination and orientation perception [84–89]. Face identification is also considered a largely perceptual process in the sense that recognition involves perceiving invariant structures of the face [90]. Furthermore, the processing of these unchangeable aspects of faces (e.g., identification) seems to be behaviorally and neurally distinct from processing more changeable aspects of faces, such as emotional expression and eye gaze [91, 92]. These more changeable aspects of faces provide the foundation for social communication, and the perception of them is arguably a more highly developed skill compared to face identification [90]. Thus, our findings suggest that at the most basic perceptual level, face processing, including both where individuals initially orient their gaze and the resulting rapid identification/recognition, may be largely intact in ASD.

### Other aspects of face identification are impaired in ASD

In the context of intact initial eye movements to faces and rapid face identification, we found that adolescents with ASD performed significantly worse compared to TD controls on a more traditional measure of face identification, the DFPT. The DFPT, which has not previously been used in ASD, was initially developed for use in patients with developmental prosopagnosia as a measure of face perception, to be compared against measures of face memory, in order to better dissociate the processes of face perception and face memory in this population [75]. Children ages 7 to 12 with developmental prosopagnosia have been shown to perform around chance on this measure, highlighting their substantial difficulties with face perception (in addition to face memory as measured by other tasks [75];). Compared to children with developmental prosopagnosia, the adolescents with ASD from the current study performed, on average, far above chance suggesting that ASD is not characterized by face perception deficits as severe as developmental prosopagnosia.

Our finding of differential performance across the two face identification tasks provides an opportunity to dissociate which aspects of face identification may or may not be impaired in ASD. Although there was a moderate correlation between performance on these two tasks, a finding consistent with the observation that the tasks were similar in many ways (e.g., alternative forced-choice task, gray scale face stimuli showing only the face and not hair/ears/body), the group difference on the DFPT persisted when controlling for face identification performance on the free viewing first look face task. This leads to greater confidence that the deficits in performance in ASD can be attributed to the unique components of the DFPT that were not shared by the face identification component of the free viewing task. Specifically, the DFPT differed from the face identification component of the free viewing task in several ways. In addition to some minor differences between these tasks (i.e., three versus four response choice, child versus adult faces), several more substantive differences may help inform which specific aspects of face identification may be impaired in ASD.

First, compared to the free viewing task, which measured face identification abilities using perceptually identical target and choice faces (i.e., the face viewed in the stimulus presentation stage was an identical match to one of the four choices in the response stage), the DFPT measured face identification abilities with more perceptually complex stimuli. Specifically, the choice faces were morphs between the target face and another face and differed from each other in the percentage of the target face relative to the other face. Therefore, choice faces were not completely unique identities, thus increasing the perceptual demand of the task. In addition, the DFPT target face was presented at a 30° angle and the choice faces were presented looking straight forward. The combination of using morphs and varying presenting angles forces individuals to process faces more holistically because the target and choice faces are not perceptually identical. Accordingly, it may be that the face recognition system is indeed impaired in ASD but that these impairments are not evident when doing the most basic perceptual face matching tasks. This would be consistent with the existing literature that shows more consistent recognition deficits in ASD when more fine-grained perceptual discrimination is required [44, 48–52].

Second, the first-look free viewing task utilized the same four faces over the course of many trials allowing us to measure rapid face identification abilities for overlearned faces. On the other hand, the DFPT used eight target faces, each one presented on only five trials total. Thus, the DFPT measured face identification for new and unlearned identities. Although overlearned and familiar faces differ in important ways (e.g., familiar faces have an emotional component) and may not be processed in an identical manner [93], our finding of intact face identification in ASD on the task using overlearned stimuli but impaired performance on the task using more novel identities may be in line with literature showing atypical neural activation in ASD to unfamiliar [94, 95] but not to familiar faces [96].

Third, whereas the free viewing task measured rapid face identification abilities (only allowing participants to very briefly view the stimulus), the DFPT was self-paced and participants were free to take as long as they wished before selecting their response. The reaction time data from the DFPT suggest that adolescents with ASD viewed the stimuli for at least as long as the TD controls; however, their performance was still worse. It is possible that TD adolescents were able to gain more information about identity when permitted to look at the stimulus for longer than one brief moment (as in the free viewing task) but that adolescents with ASD were unable to gain as much information, compared to controls, in this untimed task.

Fourth, compared to the free viewing task, where the target face and array of choice faces were presented sequentially, the DFPT presented the target and choice faces on the screen simultaneously. Thus, the DFPT removed all working memory demands from the task of face identification. Importantly, the finding that adolescents with ASD performed worse on the task with no memory demands but similarly to TD controls on the task with some working memory demands is in contrast to the idea that face memory deficits may be a possible explanation for the mixed findings on face identification abilities in ASD across the literature [42]. Our findings suggest that, at least in the context of very brief stimulus presentation times, low levels of working memory demands do not impact face identification performance in ASD. In addition, performance deficits in ASD on a task with no memory demands suggest that perceptual complexity may instead limit performance.

In sum, it is clear that individuals with ASD show a deficit on this more traditional face identification task in the context of intact rapid face identification abilities. Our experimental design, and specifically the utilization of two different tasks that both focused on perceptual aspects of face identification, allowed us to examine more fine-grained aspects of face identification within the same group of participants. Although it is impossible to definitively determine which differing aspects of these two tasks explain our findings, we suggest perceptual complexity or temporal factors (or a combination of both) as the most likely candidates. It will be important for future research to disentangle these factors by independently investigating the effects of perceptual similarity and presentation time on face identification. The use of fMRI paradigms to characterize the neural mechanisms underlying these specific aspects of face processing may help elucidate differences in neurodevelopment in ASD.

### Possible developmental changes in initial eye gaze to faces and rapid face identification abilities

When comparing across the adolescent and adult participants, one interesting result was that face identification performance for these briefly presented faces improved with age. However, there were no relationships between age and initial eye movement location or variability. Together, this suggests that mechanisms to detect faces in the periphery and support initial orientation of eye gaze to faces may develop earlier than do separate, specialized recognition mechanisms [55]; the latter may continue to develop through late adolescence and into early adulthood. This is consistent with the idea that detection mechanisms may be hardwired at birth and that recognition mechanisms become specialized through experience [83]. Although the most substantial developmental improvements in simple face identification occur through age 11 [62–64], face recognition abilities do not appear to be fully mature until late adolescence [97, 98] or even adulthood [65, 66]. Our results extend these developmental findings to rapid face identification abilities that rely on a single moment of processing and suggest that these abilities continue to develop at least through early adulthood.

Another interesting developmental result was the lack of relationship between preferred first fixation location and performance differences at various forced fixation locations in our adolescent sample (across both the ASD and TD groups). This relationship in adults, which was found in our adult sample and in previous studies, is indicative of optimization of one’s preferred initial eye movement location on the face that maximizes performance [25, 55]. Therefore, our data begin to suggest that this optimization may develop over time and support the development of rapid face identification abilities. Future research should explore the possibility of the development of optimal initial fixations to faces and examine if a developing optimization may contribute to the honing of face identification abilities through late adolescence or even early adulthood.

One question that warrants further investigation is whether face identification abilities develop to become more rapid over time. Specifically, face identification performance in neurotypical adults has been shown to reach its maximum after two fixations, and additional viewing time does not impact performance [23]. However, it remains unknown how long a face must be viewed to maximize identification performance for children or adolescents. It is possible that our sample of adolescents was still developing their individualized optimal first fixation location and that this limited their identification abilities when only permitted to briefly view the face. It will be important for future research to investigate these possible developmental changes using both cross-sectional and longitudinal designs over wider age ranges.

Another question relates to how these possible developmental processes may impact optimal initial eye movement locations and rapid face identification abilities in ASD. Although our results suggest no differences in an adolescent sample, it is possible that the relatively late developmental time course for these abilities could lead to differences that do not become evident until adulthood. One strength of the current study was characterizing these behaviors in ASD in the specific developmental period of adolescence to minimize possible developmental differences confounding our results. Characterizing these behaviors across both adolescence and adulthood in typically developing individuals allowed us to make some preliminary conclusions about developmental trajectories of these behaviors; future work should similarly examine these trajectories in adults with ASD.

### Limitations and additional future directions

One important limitation of our study was that several adolescents with ASD were unable to complete the forced fixation condition of the main experimental task (see Additional File 1 for detailed information and supplemental analyses). This was due to an unexpectedly high number of “failed” trials as defined by the fixation break criteria. In other words, more adolescents with ASD had difficulty maintaining precise fixation prior to stimulus onset. Experimenter observations suggest that this was not due to noncompliance or reduced effort, as all participants were observed to be looking at the fixation cross. Instead, it is possible that these individuals have true difficulties maintaining fixation, even for relatively brief periods of time (500–1000 ms). Although difficulties with fixation led to a reduced sample size for the forced fixation condition, analyses for the free viewing condition were consistent across samples that displayed fixation difficulties and those that did not, suggesting that poor fixation abilities likely do not explain our results. However, future studies should systematically investigate the precision of fixation to determine if individuals with ASD have true deficits in this oculomotor ability. Some studies have shown that individuals with ASD make more saccades, reflecting shorter fixation durations, when viewing geometric shapes [99] and when performing visual search tasks [100]. Other studies have shown that fixations in ASD are not modulated by factors such as task demands [101] in a typical way. In addition, infants at risk for developing ASD (based on having an older sibling with a diagnosis of ASD) have been shown to have shorter fixation durations when viewing static images on a screen (average of 350 ms compared to 390 ms for low-risk infants [102];). These studies reflect basic differences in fixation when naturally exploring images and suggest some atypicality in balancing saccades with fixations. However, no studies to date have investigated fixation precision in ASD when the task itself is to maintain fixation. In other words, it remains unknown if individuals with ASD are simply unable to precisely maintain fixation or whether they have the capability when exerting conscious effort but display different eye movement patterns when visually exploring. Understanding how fixation abilities under conscious control might differ in ASD may help interpret studies that use paradigms where maintenance of fixation is essential to the measurement of interest (e.g., perceptual after-effects [103–106];).

Our findings speak to the sparing of the initial eye movement to faces and its resulting consequence on rapid face identification in adolescence, but it is possible that first look behaviors are atypical in the early years and normalize with development. If these processes are atypical early in ASD, they could have cascading developmental consequences, even if the initial eye movements themselves appear typical by adolescence. Thus, in addition to testing adults with ASD (as described above), it will be important to understand if there are divergent developmental trajectories in initial face detection and orientation between ASD and TD, and if so, how these differences contribute to symptoms and functioning throughout development.

While we are confident in our set of results, the small sample size in this study does limit our ability to generalize our findings across the heterogeneous autism spectrum. Specifically, our sample only included one female with ASD, thus largely limiting our conclusions to males with ASD. In addition, it is possible that individuals with high functioning ASD, as assessed in the current study, demonstrate intact initial eye gaze to faces but that individuals with more severe functional impairments show differences in these first look behaviors.

To test these behaviors in those with lower cognitive abilities and in younger individuals, this paradigm will need to be significantly further adapted. The process used in the current study to adapt the paradigm for use in adolescents with and without ASD (see Additional file 1) can be used as an example of how to successfully adapt existing paradigms for different populations. Although the focus of the current study allowed us to maintain the experimental rigor to best compare our findings with previous literature, future adaptations of the paradigm will likely require further considerations of how to best weigh experimental stringency with practical limitations. Specifically, younger and lower functioning individuals will likely be unable to maintain fixation and make a behavioral response. Thus, adaptations for these populations could focus on analyzing each fixation on a moment by moment basis in a more passive viewing paradigm. Electroencephalography (EEG) may also be a fruitful method to consider in combination with eye tracking, given that passive EEG paradigms have successfully been applied in these populations (e.g., [107]).

## Summary and conclusion

In summary, this study allowed us to carefully and comprehensively investigate a single moment of processing (the initial eye movement to a face) in the very complex process of face perception in adolescents with and without ASD, in addition to neurotypical adults. The findings indicated that adolescents with and without ASD show remarkable similarities in their initial eye movements to faces and rapid face identification abilities, which suggest intact detection and initial orientation to and rapid recognition of faces in this population. However, adolescents with ASD showed worse performance on a more perceptually demanding and time-unlimited face identification task, suggesting deficits in face identification more downstream of the initial eye movement. This strongly supports a need to investigate face processing on a moment-by-moment basis to understand where perceptual and attentional differences in face processing may begin to contribute to social deficits in ASD. Finally, an age-related analysis across all three participant groups revealed preliminary evidence that individualized optimal initial eye movement locations to faces may be a developmental process that continues into late adolescence or even early adulthood.

## Supplementary information

**Additional file 1.** Supplemental Material.

## Data Availability

The datasets analyzed during the current study are available from the corresponding author on reasonable request.

## Abbreviations

AQ: Autism Quotient
ASD: Autism spectrum disorder
DFPT: Dartmouth Face Perception Test
TD: Typically developing
