# Supplementary material for: Initial eye gaze to faces and its functional consequence on face identification abilities in autism spectrum disorder
Source: J Neurodev Disord. 2019 Dec 28;11:42. doi: 10.1186/s11689-019-9303-z (PMC6935487; doi:10.1186/s11689-019-9303-z)
Supplement: Supplementary file 1 — Additional file 1. Supplemental Material. [file 11689_2019_9303_MOESM1_ESM.docx]

## Additional File 1; Supplemental Material

1. **Comparison of ASD participants that did and did not complete forced fixation task.**

Several participants did not complete the forced fixation task. In the neurotypical adult group, this was due to two participants not returning to complete day two of the study. In the adolescent group, this was due to time constraints that prevented completion of all experimental tasks. Specifically, as designed, both the free viewing and forced fixation tasks needed to be completed in the same experimental session in order to reduce face memory demands. For most participants, this was possible. However, some participants (8 ASD, 1 TD) required more frequent breaks and/or had greater difficulty with eye-tracking calibration and maintaining fixation, leading to a significantly longer task duration. When this occurred, quality of data for the free viewing task and collection of the control task were prioritized over collection of the forced fixation task.

Supplemental analyses were conducted to examine how our results may have been influenced by those adolescents who were unable to complete all tasks. Free viewing analyses were repeated in two subsamples. First, to ensure that those who were unable to complete the forced fixation task did not comprise a unique subpopulation, all free viewing task analyses were repeated including only those participants who also completed the forced fixation task. Second, to ensure that these results could not be explained by methodological adjustments made to accommodate participants who had difficulty with fixation maintenance, analyses were repeated including only those who had a tolerance setting of 1.5° of visual angle from the center of the fixation cross. Of note, many of the participants who did not complete the forced fixation task overlapped with those who required tolerance adjustments. In these two subsamples, all results were consistent with the results reported in the main text with the full sample.

In addition, given the reduced sample size for the forced fixation task, we conducted a post-hoc Bayes factor analysis on the effect of group (ASD vs. TD adolescents comparison of face identification accuracy collapsed across forced fixation locations; *B* = 0.09), which strongly supported the null effect and suggested that this result was not likely due to a lack of power to detect a difference. Finally, participants with ASD who were able to complete the forced fixation task (*n* = 13) were directly compared to those who were not (*n* = 8) on several demographic, diagnostic, and free viewing task experimental variables. There were no significant differences in age, (completed task: 14.29 (1.37), did not complete task: 13.82 (1.65), *t*(19) = .71, *p* = .49); in FSIQ, (completed task: 108.15 (10.83), did not complete task: 98.63 (11.64), *t*(19) = 1.90, *p* = .07); or in the ADOS-2 calibrated severity score, (completed task: 6.15 (1.68), did not complete task: 7.00 (1.60), *t*(19) = 1.14, *p* = .27). There were also no differences in the average landing location of the initial eye movement in either the horizontal or vertical dimension (all *p* > .40). However, those who were not able to complete the forced fixation task demonstrated greater variability (standard deviation) in their landing locations across trials (horizontal dimension: *t*(19) = 2.29, *p* = .03, *d* = 1.02; vertical dimension: *t*(8.43) = 2.04, *p* = .07, *d* = .99), and lower face identification performance, *t*(19) = -2.19, *p* = .04, *d* = 1.01. Given the non-significant trend towards an effect of IQ, correlations between FSIQ and eye tracking measures across the full ASD sample were conducted to ensure that our paradigm was not significantly impacted by cognitive ability. All correlations with FSIQ were non-significant: mean landing location of initial eye movement in the vertical dimension (*r*(19) = -.17, *p* = .47), standard deviation of initial eye movement in the vertical dimension (*r*(19) = -.29, *p* = .20), mean landing location of initial eye movement in the horizontal dimension (*r*(19) = -.30, *p* = .18), standard deviation of initial eye movement in the horizontal dimension (*r*(19) = .08, *p* = .72).

This set of analyses further supports our main conclusions that adolescents with and without ASD show similar patterns of initial eye gaze to faces and rapid face identification abilities. However, one important limitation of our study was that several adolescents with ASD were unable to complete the forced fixation condition of the main experimental task. This was due to an unexpectedly high number of “failed” trials as defined by the fixation break criteria. In other words, more adolescents with ASD had difficulty maintaining precise fixation prior to stimulus onset. Experimenter observations suggest that this was not due to noncompliance or reduced effort, as all participants were observed to be looking at the fixation cross. Instead, it is possible that these individuals have true difficulties maintaining fixation, even for relatively brief periods of time (500-1000ms). See the main text for an additional discussion of this limitation.

1. **Control Task: Free Viewing of Briefly Presented Snowflakes**

All adolescent participants who completed the free viewing task with briefly presented faces also completed this control task. The purpose of this task was to control for possible confounds, including group differences in our adolescent samples in task motivation and basic abilities to make a saccade to briefly presented stimuli more generally. Although a recent meta-analysis concluded that visually guided (reflexive) saccades are largely intact in ASD, with no group differences in saccade latencies, peak velocity, and mean accuracy [9], there is some evidence that oculomotor functioning is impaired, particularly when the task places high demands on the motor system [10, 11]. Our face identification task minimizes these demands by requiring a short saccade distance; however, incorporating this control task allowed us to account for any differences accordingly. This control task was developed and piloted in a subgroup of the adult participants (*n* = 25).

This control task was almost identical to the free viewing face identification task, except that participants were presented with a snowflake image instead of a face (Figure S.1). Specifically, stimuli were four snowflake images (same size and contrast as face stimuli) presented for 350 ms. Participants’ task in the response phase was to identify which pattern was previously presented. Participants completed 3-blocks of this control task.


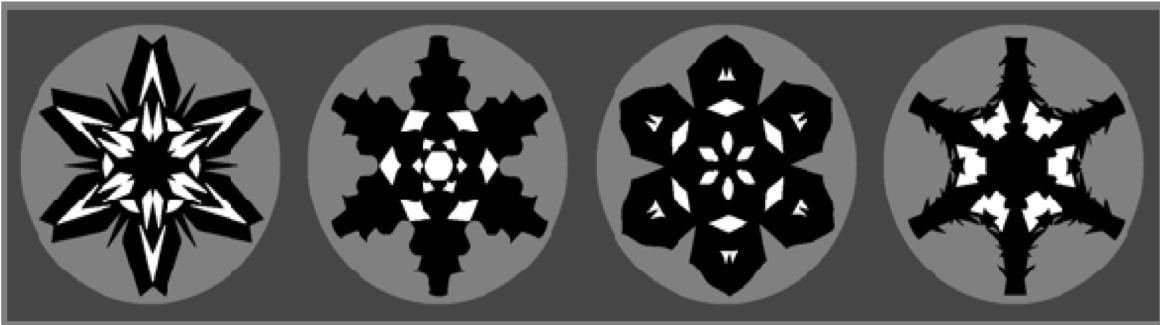


***Figure S.1.*** Snowflake stimuli used in the control task.

Data from the control task was compared between the adolescents with and without ASD to rule out possible confounding factors. After discarding trials from the first block to eliminate effects of learning this new stimulus set, blocks 2 and 3 (total of 120 trials) were analyzed for this condition. We calculated the landing location (in degrees of visual angle below the center) of the first registered saccade for each trial. Then, two variables were calculated for each participant across trials: 1) average initial eye movement location and 2) standard deviation (SD) of initial eye movement location. Percent correct identification was calculated from the participants’ responses. We then used independent samples *t*-tests to compare adolescent groups on these three variables.

When comparing the two adolescent groups, there were no differences on the snowflake control task in average initial eye movement location, *t*(40) = 1.38, *p* = .18, *d* = .43, variability of initial eye movement location, *t*(40) = .55, *p* = .59, *d* = .17, or percent correct identification, *t*(40) = 1.08, *p* = .29, *d* = .33. This suggests that adolescent groups did not differ on their general task compliance and attention, basic abilities to make a saccade to a briefly presented image, and basic perceptual discrimination abilities.

1. **Paradigm development.**

Neurotypical adults completed a longer, but otherwise identical, version of the free viewing and forced fixation conditions of the main first look face identification experimental task. This extended version included 4 blocks of task training/face familiarization, 8 blocks of free viewing, and 15 blocks of forced fixation. This data was then used to systematically evaluate the paradigm with the goal of further shortening the original paradigm as much as possible, while also maintaining a similar degree of power to preserve detection of all anticipated effects based on the existing literature using this paradigm in neurotypical adults. To do this, we first visually inspected the data to determine the point at which additional trials were no longer uniquely informative. In other words, we looked for the point where the data converged for our main variables of interest (face identification accuracy, average initial eye movement location). This process revealed that we could truncate the paradigm almost in half, yielding a 4-block (compared to 8-block) free viewing face identification paradigm and a 7-block (compared to 15-block) forced fixation paradigm. The number of trials remained consistent at 60 trials per block. We analyzed the data twice – first using all of the trials, and then using only the trials from the first 4 blocks of the free viewing face identification condition and the first 7 blocks of the forced fixation condition. Results across both versions were consistent with published data using this paradigm [12-16]. We also directly compared all main variables of interest using Pearson correlations to ensure that the shortened paradigm was appropriately similar to the full paradigm (See Table S.1).

Table S.1

Comparison of Full and Shortened Versions of the Face Paradigm

|  | Full Paradigm | | Shortened Paradigm | | |  |
| --- | --- | --- | --- | --- | --- | --- |
|  | Mean | SD | Mean | SD | Correlation Coefficient | |
| Free Viewing |  |  |  |  |  | |
| Mean X | -.56 | (.07) | -.54 | (.07) | 0.96 | |
| SD X | 1.68 | (.10) | 1.63 | (.10) | 0.97 | |
| Mean Y | 2.03 | (.24) | 2.07 | (.24) | 0.996 | |
| SD Y | 1.66 | (.08) | 1.63 | (.08) | 0.97 | |
| Percent Correct | .76 | (.02) | .76 | (.02) | 0.98 | |
|  |  |  |  |  |  | |
| Forced Fixation |  |  |  |  |  | |
| Forehead | .61 | (.03) | .61 | (.03) | 0.96 | |
| Eyes | .73 | (.03) | .73 | (.03) | 0.95 | |
| Mid Nose | .74 | (.03) | .74 | (.03) | 0.97 | |
| Tip of Nose | .73 | (.03) | .73 | (.03) | 0.97 | |
| Mouth | .61 | (.02) | .61 | (.02) | 0.96 | |
|  |  |  |  |  |  | |
| Peak Location | 1.37 | (.22) | 1.37 | (.27) | 0.93 | |
|  |  |  |  |  |  | |
| Peak Performance | .75 | (.03) | .75 | (.03) | 0.97 | |

SD = standard deviation. Correlation coefficient = R value from Pearson correlation. Peak Location and Performance are variables calculated from quadratic model fits to individuals’ forced fixation data.

All results reported in the main manuscript include only those trials that were completed across all three participant groups (i.e., the trials that were included in the shortened version of the paradigm). In addition to reducing the number of blocks per condition, the variable fixation period before stimulus onset was 500-1500 ms in the longer version, and was changed to 500-1000 ms in the shortened paradigm to reduce the fixation demand and prevent an excess of failed trials in adolescent groups.

1. **Additional analyses for the free viewing task to rule out impact of excluded trials.**

Subjects with ASD had a greater number of excluded trials compared to subjects with TD in the free viewing task. Within the TD group, the number of excluded trials ranged from 3-63 trials across all 240 trials of the free viewing task. Within the ASD group, the range of excluded trials was 4-188, with 8 subjects having more than 63 excluded trials (i.e., falling outside of the TD range). To ensure that this group difference in number of excluded trials did not impact our main findings, we repeated our main adolescent analyses by including only those subjects that had 63 or fewer trials excluded. These new ASD (*n* = 13) and TD (*n* = 21) groups did not differ on initial fixation location (*t*(32) = .68, *p* = .50), standard deviation of initial fixation location (*t*(32) = .84, *p* = .41), or percent correct identification (*t*(32) = .30, *p* = .77).

Of note, six of the eight subjects with a high number of excluded trials overlapped with the subjects that were unable to complete the forced fixation task. This overlap further supports the idea that there may be some individuals with ASD that have true difficulties in aspects of basic eye movements, including maintaining fixation (see Discussion: Limitations and Additional Future Directions).

1. **Additional analyses for free viewing/forced fixation relationship in adults and adolescents**

Our main analytic approach to test for relationships between preferred initial eye movement locations and patterns of performance on the forced fixation condition was to correlate each individual’s average first fixation location from the free viewing condition with the difference in their performance (face identification accuracy) at high (eyes) and low (mouth) forced fixation locations (see Figure 5 and Results: *Free Viewing Related to Forced Fixation*). This yielded the expected strong, positive relationship in neurotypical adults, replicating previous findings of optimal initial eye movement locations to faces, and revealed a lack of this relationship in both adolescents with and without ASD. To further explore this differential finding whereby adults show optimal initial eye movements to faces and adolescents do not, two additional analyses were each conducted separately in the neurotypical adults and adolescent groups.

First, adult participants were divided into three groups (high-lookers, mid-lookers, and low-lookers) based on their average first fixation location from the free viewing condition. Data were combined across both adolescent groups, because there were no differences on either the free viewing or forced fixation tasks based on diagnostic group in the adolescent sample (see Results: *Forced Fixation of Briefly Presented Faces*). The combined adolescent sample was divided into three new groups based on their average landing location in the free viewing task (high-, mid-, and low- lookers). Two repeated-measures ANOVAs (one in neurotypical adult sample and one in the adolescent sample) on face identification were conducted, with forced fixation location as the within participants factor and group (high-lookers, mid-lookers, and low-lookers) as the between participants factor. In the neurotypical adult sample, this analysis revealed a significant interaction between forced fixation location and group, *F*(5.77, 103.85) = 5.90, *p* < .001, $\eta_{p}^{2}$ = .25 (Figure S.2A). Follow-up ANOVAs for each forced fixation location revealed a significant group difference specifically at the forehead location, *F*(2,38) = 3.81, *p* = .03, $\eta_{p}^{2}$ = .18, with post-hoc LSD group comparisons showing that low-lookers performed significantly worse compared to high-lookers (*p* = .009). In the adolescent sample, this analysis revealed a significant effect of fixation location, *F*(4,120) = 28.5, *p* < .001, $\eta_{p}^{2}$= .49, but no effect of group, *F*(2,30) = .52, *p* = .60, $\eta_{p}^{2}$= .03, nor interaction between group and fixation location, *F*(8,120) = 1.11, *p* = .36, $\eta_{p}^{2}$= .07 (Figure S.2C). Thus, patterns of performance at various locations on the face did not depend on where adolescents naturally initially looked to a face, unlike what we observed in neurotypical adults.

Second, to directly examine relationships between free viewing and forced fixation variables, a quadratic function was fit to each individual’s performance across the forced fixation locations. The maximum value was used to identify peak performance. A paired *t*-test was conducted between this peak performance value and individuals’ free viewing performance to determine whether individuals were maximizing their performance during the free viewing condition. In the neurotypical adult sample, peak performance did not differ from individuals’ free viewing face identification accuracy (paired *t*-test, *t*(37) = .27, *p* = .79, *d* = .04). However, in the adolescent sample, performance was better at their peak in the forced fixation condition compared to their free viewing face identification accuracy (*t*(32) = 3.16, *p* = .003, *d* = .55). Furthermore, the face location (in degrees below the eyes) that corresponded with each individual’s peak performance was entered into a correlation with each individual’s average look location from the free viewing condition. In the neurotypical adult sample, these values were strongly correlated, *r*(37) = .63, *p* < .001 (Figure S.2B), whereas in the adolescent samples, they were not, ASD: *r*(11) = .36, *p* = .23; TD: *r*(18) = .14, *p* = .55; combined: *r*(31) = .25, *p* = .16 (Figure S.2D).

Together, these supplemental analyses are consistent with the results from our primary analysis method reported in the manuscript and support our conclusions whereby performance on simple face related tasks is maximized when making an initial eye movement to one’s preferred location in neurotypical adults. However, face identification abilities may not be maximized when adolescents both with and without ASD naturally freely view faces.


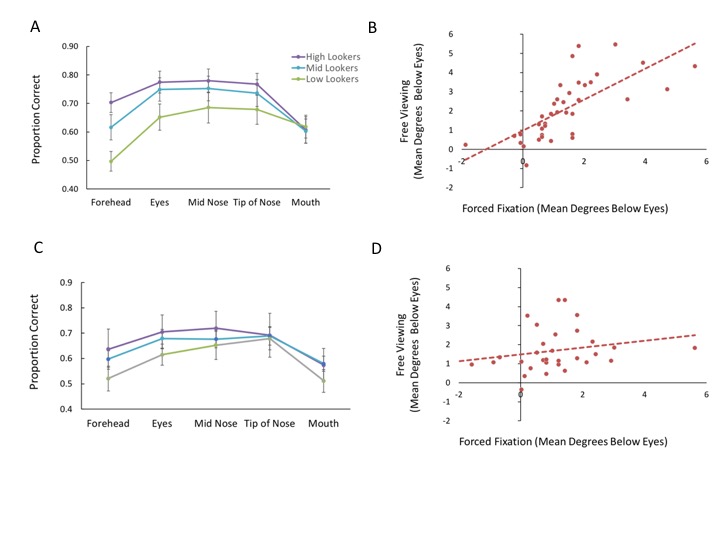


***Figure S.2.*** Supplemental analyses comparing free viewing and forced fixation performance in adults (A and B) and adolescents (C and D). A and C) Patterns of performance on the forced fixation task vary depending on their looking group, as defined by their average initial eye movement location from the free viewing task. B and D) Average initial eye movement location (measured in degrees below the eyes; y-axis) from the free viewing task is related to the location that corresponded to peak performance in the forced fixation task (x-axis), identified through quadratic model fits at the individual level. The strong correlation observed in adults, (*r*(37) = .71, *p* < .001), but not adolescents, *r*(31) = .25, *p* = .16, complements the findings reported in the main manuscript (between average initial eye movement location from the viewing task and the difference in performance between the eye and mouth locations in the forced fixation task) and indicates that individual variability in natural initial eye movement locations is related to face identification performance only in adults whereby adults, but not adolescents, naturally maximize their face identification abilities by initially looking to their own individual optimal location.

1. Kemner C, Verbaten MN, Cuperus JM, Camfferman G, van Engeland H. Abnormal saccadic eye movements in autistic children. *J Autism Dev Disord* 1998;28**:**61-67.

2. Joseph RM, Keehn B, Connolly C, Wolfe JM, Horowitz TS. Why is visual search superior in autism spectrum disorder? *Dev Sci* 2009;12**:**1083-1096.

3. Benson V, Piper J, Fletcher-Watson S. Atypical saccadic scanning in autistic spectrum disorder. *Neuropsychologia* 2009;47**:**1178-1182.

4. Wass SV, Jones EJ, Gliga T, Smith TJ, Charman T, Johnson MH, team B. Shorter spontaneous fixation durations in infants with later emerging autism. *Sci Rep* 2015;5**:**8284.

5. Rutherford MD, Troubridge EK, Walsh J. Visual afterimages of emotional faces in high functioning autism. *J Autism Dev Disord* 2012;42**:**221-229.

6. Pellicano E, Jeffery L, Burr D, Rhodes G. Abnormal adaptive face-coding mechanisms in children with autism spectrum disorder. *Curr Biol* 2007;17**:**1508-1512.

7. Ewing L, Leach K, Pellicano E, Jeffery L, Rhodes G. Reduced face aftereffects in autism are not due to poor attention. *PLoS One* 2013;8**:**e81353.

8. Cook J, Swapp D, Pan X, Bianchi-Berthouze N, Blakemore SJ. Atypical interference effect of action observation in autism spectrum conditions. *Psychol Med* 2014;44**:**731-740.

9. Johnson BP, Lum JA, Rinehart NJ, Fielding J. Ocular motor disturbances in autism spectrum disorders: Systematic review and comprehensive meta-analysis. *Neurosci Biobehav Rev* 2016;69**:**260-279.

10. Schmitt LM, Cook EH, Sweeney JA, Mosconi MW. Saccadic eye movement abnormalities in autism spectrum disorder indicate dysfunctions in cerebellum and brainstem. *Mol Autism* 2014;5**:**47.

11. Minshew NJ, Luna B, Sweeney JA. Oculomotor evidence for neocortical systems but not cerebellar dysfunction in autism. *Neurology* 1999;52**:**917-922.

12. Or CC, Peterson MF, Eckstein MP. Initial eye movements during face identification are optimal and similar across cultures. *J Vis* 2015;15**:**12.

13. Peterson MF, Eckstein MP. Looking just below the eyes is optimal across face recognition tasks. *Proc Natl Acad Sci U S A* 2012;109**:**E3314-3323.

14. Peterson MF, Eckstein MP. Individual differences in eye movements during face identification reflect observer-specific optimal points of fixation. *Psychol Sci* 2013;24**:**1216-1225.

15. Peterson MF, Lin J, Zaun I, Kanwisher N. Individual differences in face-looking behavior generalize from the lab to the world. *J Vis* 2016;16**:**12.

16. Tsank Y, Eckstein MP. Domain Specificity of Oculomotor Learning after Changes in Sensory Processing. *J Neurosci* 2017;37**:**11469-11484.
